# Investigating the Genetic and Molecular Basis of Melanin and Edible Quality in *Auricularia cornea*

**DOI:** 10.3390/jof12060381

**Published:** 2026-05-23

**Authors:** Yuling Cui, Fangjie Yao, Xiaoxu Ma, Tingting Liu, Xu Sun, Ming Fang, Lixin Lu, Youmin Zhang, Yinghao Li, Xinming Chen, Xinyue Xu

**Affiliations:** 1 Engineering Research Center of Edible and Medicinal Fungi, Ministry of Education, Jilin Agricultural University, Changchun 130118, China; cuiyuling5906@163.com (Y.C.); sunxu0512@163.com (X.S.); 2 Laboratory of the Genetic Breeding of Edible Mushroom, College of Horticulture, Jilin Agricultural University, Changchun 130118, China; maxiaoxu54@163.com (X.M.); fangming@jlau.edu.cn (M.F.); lixinl@jlau.edu.cn (L.L.); zhangymf@aliyun.com (Y.Z.); 15246928761@163.com (Y.L.); 18084220398@163.com (X.C.); 13894808630@163.com (X.X.); 3 School of Food Science and Engineering, Jilin Agricultural University, Changchun 130118, China; liutingting@jlau.edu.cn

**Keywords:** *Auricularia cornea*, melanin, edible quality, cell wall, genome-wide association study

## Abstract

For the first time, a regulatory network linking melanin, genes, pathways, and edible quality was constructed for 138 *Auricularia cornea* strains sourced domestically and internationally. This marks the inaugural study of *A. cornea* spanning from cellular to physical-mechanical properties. Correlation analysis between melanin and edible quality traits (hardness, springiness, cohesiveness, gumminess, chewiness, and resilience) revealed that hardness, cohesiveness, and gumminess increased with rising melanin content, while springiness correspondingly decreased. Genome-wide association analysis identified 15,597,589 SNP loci. A total of 39 genes related to food quality were annotated, including one melanin-related lacquer enzyme gene, *ACW004924*. Real-time quantitative PCR validation of key genes identified for melanin and edible quality traits revealed results consistent with those from correlation analysis. The lacquer enzyme genes *ACW004736*, *ACW006232*, which regulate melanin synthesis, and the tyrosinase genes *ACW001451*, *ACW002443*, and *ACW001003* were also identified in edible quality traits. These genes perform similar functions in GO-enriched metabolic processes, catalytic activity, and cellular structural complexes, as well as in KEGG-enriched pathways such as carbon metabolism and polysaccharide synthesis. They catalyze melanin synthesis and promote interactions between melanin and cell wall polysaccharides, chitin, and structural proteins, thereby stabilizing the cellular scaffold structure, jointly mediating the effect of melanin on the edible quality of *A. cornea*. The results supplement the downstream regulatory chain of catalytic enzymes and edible quality in the γ-L-glutaminyl-3,4-dihydroxybenzene (GDHB) pigment synthesis pathway, and form an information network of melanin synthesis, cell wall structure optimization, and edible quality regulation.

## 1. Introduction

**Figure 1:**
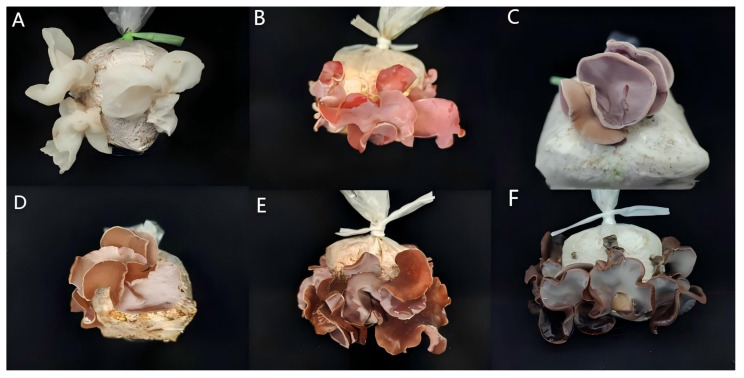
Diagram showing the emergence of *A. cornea* in different colors. (**A**) White. (**B**) Pink. (**C**) Purple. (**D**) Light brown. (**E**) Reddish brown. (**F**) Dark reddish brown.

*A. cornea* is a fungus belonging to the Auriculariaceae family and the *Auricularia* genus. It possesses rich nutritional value and contains various active components such as melanin, polysaccharides, and proteins [1]. With growing health-conscious demands for food quality, consumers now seek not only high nutritional content but also appealing appearance and texture. Color is a crucial commercial trait for *A. cornea*, where melanin serves both as an antioxidant and as the pigment responsible for its color. Extracting melanin from *A. cornea* as a raw material offers low-cost, readily available sources and a safe, healthy origin, presenting potential for development as a natural melanin source. The domestication and breeding of white-fleshed *A. cornea* mutants and naturally occurring pink *A. cornea* not only enriches China’s *A. cornea* germplasm diversity but also broadens the *A. cornea* consumer market. Currently, *A. cornea* fruit colors primarily include white, pink, purple, light brown, reddish brown, and dark reddish brown (Figure 1). The team previously obtained an *A. cornea* genome with 13 chromosomes, inferring that *A. cornea* pigments are synthesized through the GDHB pigment pathway as a single pigment type, and clarified the synthesis pathway of *A. cornea* melanin (Figure S1) [2]. Furthermore, it was found that the pectin content in *A. cornea* is negatively correlated with melanin levels—the higher the pectin content, the lower the melanin content [3]. Gum content is typically associated with edible quality traits of mushrooms (hardness, springiness, cohesiveness, gumminess, chewiness, and resilience). Our definitions of these edible quality indicators are derived from the relevant research by Alina Surmacka Szczesniak [4]. However, the underlying mechanism linking *A. cornea* melanin to edible quality remains unclear, which has, to some extent, hindered the progress of *A. cornea* variety improvement. Therefore, investigating the mechanism of interaction between *A. cornea* melanin and edible quality holds significant importance for advancing *A. cornea* breeding efforts.

Melanin is a class of large-molecule hydrophobic polymers oxidized from catechol and indoleacetic acid within phenolic compounds. It exists in animals, plants, and microorganisms, and consists of black, brown, dark brown, red, or brown pigments [5]. Melanin accumulates within the cell wall, a dynamic and plastic organelle that enables fungi to alter their macroscopic morphology in response to changes in their life cycle, environmental conditions, and immune defenses. Two primary types of melanin have been identified in fungal cell walls: *Aspergillus* species contain 1,8-dihydroxynaphthalene (DHN) melanin, while Cryptococcus neoformans harbors 3,4-dihydroxyphenylalanine (DOPA) melanin. In *Cryptococcus neoformans*, a human fungal pathogen, melanin function is predicted to enhance cell wall rigidity. The inner walls of many fungal spores contain complex amorphous polymeric phenolic compounds containing melanin, which may also enhance protective effects for fungal species [6,7]. Other studies have also demonstrated that chitin and cell wall polysaccharides co-assemble into fibrous microfibrils, forming basket-like scaffolds around cells, with melanin being implicated in this process [8]. When developing biomaterials using *A. cornea* mycelium, it was discovered that the thickness and diameter of the mycelium cell walls determine the springiness and hardness of the entire mycelium composite material [9]. A study on pigments in different colored oyster mushrooms revealed that melanin granules accumulate more densely in the cell walls of darker mushrooms, which also exhibit thicker cell walls. It was further discovered that the varying colors of mushroom fruiting bodies result from differences in the types, proportions, and quantities of melanin present [10]. Extracting pigments from plants as natural food colorants and antioxidants effectively reduces food hardness and chewiness, making the texture more appealing to consumers [11]. Another study found that the edible quality of *Auricularia heimuer* is related to its content of total sugars, crude fiber, crude polysaccharides, protein, and amino acids [12]. Other studies have also demonstrated that the addition of nutritional bioactive compounds from *A. cornea* significantly enhances the hardness and cohesiveness of food products [13].

Genome-wide association studies (GWAS) enable the identification of significant gene-phenotype associations across the entire genome in large-scale samples. Genetic markers across the entire genome were detected in multiple individuals, genotypes were obtained, and population-level statistical analyses linking genotypes to phenotypes were conducted. Based on statistical significance, the genetic markers most likely to influence the trait were identified. Subsequently, genes associated with trait variation were uncovered by leveraging the linkage disequilibrium between markers and functional genes [14]. GWAS enables the identification of significant gene-phenotype associations across the entire genome in large-scale samples. Characterized by high efficiency and accuracy, they are widely applied in crop research [15,16]. Research on edible fungi has also been initiated gradually. Zhang et al. [17] conducted a genome-wide association study (GWAS) of 133 shiitake mushroom samples, identifying genes associated with fruiting body development and elucidating the genetic basis of shiitake mushroom evolution. Qian et al. conducted genetic diversity analysis on 47 collected oyster mushroom strains using GWAS and constructed fingerprint profiles [18]. Traditional breeding methods rely solely on phenotypic selection, whereas GWAS enables fundamental precision breeding. To date, no GWAS-based studies have been reported for *A. cornea*. This research represents the first genome-wide association analysis of nutritional and edible qualities in *A. cornea*, identifying genes associated with target traits. This approach facilitates marker-assisted breeding, accelerates quality improvement in *A. cornea* cultivation, and meets diverse market demands.

The team previously identified the synthesis pathway of pigments in *A. cornea* and discovered a correlation between their gelatinous substance and melanin. However, the fruiting bodies of *A. cornea* exhibit a wide range of colors, and the edible quality varies significantly among different colors. To date, the relationship and underlying mechanisms between melanin content and edible quality in *A. cornea* remain unclear, with no related research reports available. The team assisted in genome assembly through Hi-C in the early stage. A single genome consisting of 13 chromosomes was obtained [2]. The genome data have been published and can be obtained from the NCBI database (https://www.ncbi.nlm.nih.gov/bioproject/PRJNA943604/, accessed on 20 March 2026). This study employs GWAS to analyze the association between *A. cornea* melanin and edible quality. This approach aims to identify relevant genes and clarify their functions, holding significant implications for *A. cornea* variety improvement and breeding.

## 2. Materials and Methods

### 2.1. Strain Collection and Preservation

A total of 138 *A. cornea* strains (103 wild strains, 29 cultivated strains, and 6 hybrid strains) were collected from Lusaka, Zambia, Africa; Kyoto City, Hikone City, and Okinawa Prefecture, Japan; Hamilton, New Zealand; and 16 provinces in China (Table S1). For long-term preservation, mycelial plugs were transferred to sterile cryovials containing 20% glycerol and stored at −80 °C. All strains were deposited at the College of Horticulture, Jilin Agricultural University.

### 2.2. Melanin Extraction and Content Determination

Fruiting bodies of *A. cornea* in various colors were collected and air-dried naturally. The dried fruiting bodies were ground using a food processor or grinder. The powdered fruiting bodies were mixed with 1.5 mol/L NaOH (Tianjin Tianli Chemical Reagent Co., Ltd., Tianjin, China) solution at a ratio of 3 g powder to 1.5 mol/L solution (1:30). The mixture was suspended in an ultrasonicator (Thermo Fisher Scientific, Carlsbad, CA, USA) at 160 W for 90 min at 25 °C. The suspension was then centrifuged at 12,000 rpm for 20 min, and the supernatant was transferred to another beaker and adjusted to pH 1.5 using 7 mol/L HCl (Tianjin Tianli Chemical Reagent Co., Ltd., Tianjin, China). The pH-adjusted supernatant was allowed to precipitate at room temperature for 3 h. Subsequently, the precipitate was collected by centrifuging at 12,000 rpm for 20 min. It was rinsed repeatedly with deionized water until the pH became neutral. The precipitate was freeze-dried to obtain crude black pigment from *A. cornea*. To obtain pure black pigment, the collected crude pigment was redissolved in 7 mol/L HCl, heated in a water bath at 100 °C for 2 h, and then centrifuged at 12,000 rpm for 20 min. The precipitate was repeatedly washed with deionized water; the washings were discarded, and it was then washed sequentially with anhydrous ethanol (Jilin Jintai Chemical Glass Co., Ltd., Changchun, China), chloroform (Jilin Jintai Chemical Glass Co., Ltd., China), and ethyl acetate (Jilin Jintai Chemical Glass Co., Ltd., China). The precipitate was air-dried at room temperature. The dried precipitate was redissolved in 2 mol/L NaOH, centrifuged at 12,000 rpm for 20 min, and the supernatant was collected. The supernatant was adjusted to pH 1.5 using 7 mol/L HCl, and the precipitate was collected and repeatedly washed with deionized water until neutral. The precipitate was dried in a 60 °C oven (China Greda Electronic Equipment Co., Ltd., Suzhou, China) to obtain pure melanin. (Note: The repeated sentence was merged.) The melanin content was determined at 450 nm wavelength using a microplate reader (with 0.1 mol/L NaOH as the blank control) [19]. Each sample was tested in triplicate.

### 2.3. Food Quality Testing

Dried *A. cornea* fruiting bodies were soaked for 4 h and then drained thoroughly. Smooth, flat, and uniformly thick earpieces were selected. From each earpiece, a 2 cm × 2 cm section was cut from the same location for later use. The edible quality of the *A. cornea* pieces was measured using the Texture Analyzer’s TPA mode (Texture analyzer from: Saicheng Electronic Technology Co., Ltd., Jinan, China). The testing conditions were set as follows: probe: P5S; pre-test speed: 1 mm/s; test speed: 2 mm/s; post-test speed: 1 mm/s; test distance: 2 mm; trigger force: Auto-5 g; zeroing mode: Auto; data acquisition rate: 400 pps. Six indicators (hardness, springiness, cohesiveness, gumminess, chewiness, and resilience) were measured for each sample [4]. Each sample was tested in triplicate.

### 2.4. Genomic DNA Extraction, Library Preparation, and Sequencing

The mycelium of *A. cornea* was collected, and genomic DNA was extracted using the cetyltrimethylammonium bromide (CTAB) method [20]. DNA quality, concentration, and purity were assessed using 1% agarose gel electrophoresis and a UV spectrophotometer, respectively. The samples were diluted to 1 ng/μL with sterile water for subsequent use. One microgram of sample DNA was sonicated using a Covaris M220 ultrasonicator to fragment the DNA into 300–500 bp fragments. For library preparation, end-blunting, 3′-A-tailing, index adapter ligation, purification, and PCR amplification were performed. After library enrichment, PCR amplification was carried out for 8 cycles. Subsequently, the target band was recovered via 2% agarose gel electrophoresis. Quantification was performed using TBS380 (Picogreen). The samples were mixed proportionally based on the data and subjected to bridge PCR amplification on cBot solid-phase carriers to generate clusters. Finally, 2 × 150 bp sequencing was performed on the Illumina HiSeq 6000 platform. This portion of the work was entrusted to Shanghai Ling’en Biotechnology Co., Ltd. for completion. The raw sequencing reads have been submitted to the NCBI Sequence Read Archive (SRA) under accession number PRJNA1406526.

### 2.5. Genome-Wide Association Study

GWAS mixed linear models (LMM) were computed using GEMMA (v0.98.1), enabling precise and rapid analysis of single-SNP GWAS, multi-SNP GWAS, and multi-trait GWAS [21]. Based on the GWAS results, loci with LOD scores > 5 were identified as significantly associated. After gene localization, enrichment analysis was performed using the COG, GO, and KEGG databases.

Candidate genes associated with GWAS were analyzed using sequence similarity searches performed using the BLASTP algorithm of the BLAST 2.14.0 [22] (Camacho et al., 2009) against the NCBI non-redundant (nr) protein database [23]. Searches were conducted with an E-value threshold of 1 × 10^−5^. Conserved domain predictions were performed using the NCBI Conserved Domain Database (CDD) [24].

### 2.6. COG, GO, and KEGG Enrichment Analysis

The whole-genome protein sequences of the hairy wood ear fungus were aligned against the EggNOG V5.0 database using EggNOG-mapper software (http://eggnog5.embl.de/) [25]. Based on the best match results, all genes were annotated with COG functional classifications. Using all annotated genes as the background reference set and the target gene set as the test set, hypergeometric distribution tests were performed with the phyper function in R (version 4.2.2) to assess whether target genes were significantly overrepresented across the 26 COG functional categories. The resulting *p*-values were corrected for multiple comparisons using the Benjamini–Hochberg method to control the false discovery rate. COG categories with corrected *p*-values (FDR) < 0.05 were identified as significantly enriched [26].

GO enrichment analysis was performed using GOATOOLS with Fisher’s exact test [27]. To control the false positive rate, *p*-values were corrected using four multiple testing methods: Bonferroni, Holm, Šidák, and false discovery rate (FDR). GO terms with corrected *p*-value (p_fdr) ≤ 0.05 were considered significantly enriched [28].

KEGG pathway enrichment analysis was performed using KOBAS-i with Fisher’s exact test [29]. Multiple testing correction was performed using the Benjamini–Hochberg method (FDR), with the significance threshold set at *p* < 0.05. KEGG pathways meeting this criterion were considered significantly enriched in the differentially expressed genes [30].

### 2.7. Statistical Analysis

All phenotypic data were exported to an Excel spreadsheet and analyzed using R version 4.2.2 [31]. The raw data of all phenotypes are provided for reference (Table S2). Principal component analysis was performed using the prcomp function with the parameters center = TRUE and scale = TRUE, based on the standardized values (z-scores) of each trait. The scores of the first two principal components were visualized as a scatter plot using ggplot2 [32] to demonstrate the natural clustering of samples. Descriptive statistics, including sample size, mean, standard deviation, range, skewness, and kurtosis for each trait, were calculated using the describe function from the psych packag. Correlation analysis was conducted using the cor. test function with method = “pearson” to calculate Pearson correlation coefficients between melanin content and each edible quality index, followed by significance testing. All *p*-values were two-sided.

### 2.8. Gene Function Validation

Four *A. cornea* strains exhibiting significant variations in melanin content and edible quality were selected: white ACW008, pink ACP126, purple ACP004, and dark reddish-brown ACP022. Six key genes controlling melanin and edible quality were identified: *ACW004924* (laccase), *ACW016160* (expansion-like protein), *ACW006238* (chitin synthase), *ACW014592* (small GTPase), *ACW011186* (keratinase), and *ACW001451* (tyrosinase-related protein). Primers were designed and validated based on the target gene sequences. The β-tubulin gene (β-TUB) was selected as the internal reference gene. Total RNA was extracted from *A. cornea* using the TRIzol Universal Kit (DP424), and cDNA was synthesized using oligo (dT) primers (detailed experimental procedures are provided in Supplementary Materials File S1). The main instruments and equipment used are listed in Table 1. The ChamQ Universal SYBR qPCR Master Mix (SYBR Green system, 20 μL) was employed. The reaction protocol was as follows: pre-denaturation at 95 °C for 30 s (1 cycle); followed by 40 cycles of denaturation at 95 °C for 10 s, annealing at 60 °C for 30 s, and denaturation at 95 °C for 15 s. Melting curve acquisition was performed at 60 °C for 60 s, 95 °C for 30 s, and 95 °C for 15 s (1 cycle). Relative quantification was carried out using the 2^−ΔΔCt^ method [33].

## 3. Results and Analysis

### 3.1. Phenotypic Assessment

**Figure 2:**
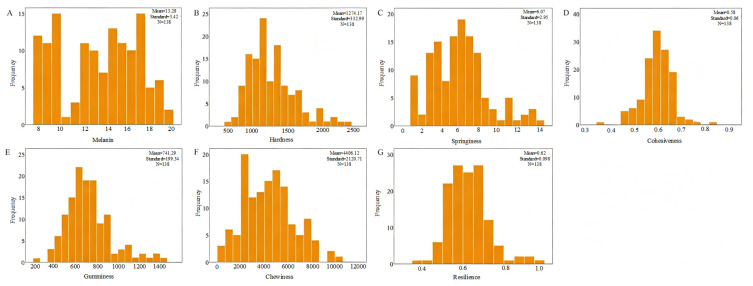
(**A**) Melanin. (**B**) Hardness. (**C**) Springiness. (**D**) Cohesiveness. (**E**) Gumminess. (**F**) Chewiness. (**G**) Resilience. Frequency distribution chart of melanin content and edible quality traits in *A. cornea*. Error bars represent the standard deviation (SD). The horizontal axis represents measured trait values, while the vertical axis indicates frequency. Higher frequency values correspond to larger sample sizes within that trait range. The melanin content of most samples is high, the distribution is right skewed, the hardness, springiness, cohesiveness, gumminess, chewiness, and resilience are at the medium level, and the distribution is symmetrical.

Analysis of 138 samples of *A. cornea* for melanin content and edible quality traits revealed the following results (Table 2): Melanin content ranged from 7.51 ng/g to 19.31 ng/g with a coefficient of variation (CV) of 26%. Hardness values ranged from 673.62 to 2342.33 with a CV of 26%. Cohesiveness ranged from 0.34 to 0.81 with a CV of 11%. Springiness ranged from 0.94 to 14.42, with a coefficient of variation of 49%. Gumminess ranged from 297.13 to 1421.02, with a coefficient of variation of 27%. Chewiness ranged from 584.72 to 10,266.32 with a coefficient of variation of 48%, and resilience ranged from 0.39 to 0.98 with a coefficient of variation of 16%. These seven traits exhibited continuous variation. The Kolmogorov–Smirnov (K-S) test indicated that the *p*-values for all traits were greater than 0.05 (Figure 2), suggesting no significant departure from normality. However, we acknowledge that the K-S test may have limitations with moderate sample sizes, and for some traits like melanin, the distribution appeared roughly bimodal despite the non-significant result. Therefore, these findings are interpreted as the data not severely violating the assumption of normality, rather than as definitive proof of a normal distribution. The white strain in this study exhibited the lowest melanin content (7.51 ng/g) and significantly reduced edible quality indicators such as hardness and gumminess. In contrast, Wang et al. [34] reported that the white mutant strain of *A. cornea* exhibited white fruiting bodies due to the deletion of a key enzyme gene from the color control locus in the synthesis pathway. However, melanin content analysis in this study revealed that white *A. cornea* does contain melanin, albeit at low levels. This discrepancy may be attributed to the use of dried *A. cornea* samples for melanin detection. During natural air-drying, polyphenol oxidase likely oxidized the fruiting bodies to a pale yellow hue. Chen et al. [35] confirmed this in their study on browning in white *A. cornea*. Furthermore, melanin color varies depending on elemental composition. For instance, eumelanin is typically black, but it can also be yellow, brown, or black, while pheomelanin ranges from yellow-brown to brown to black [36]. Subsequent analysis can be conducted to identify the types of melanin present in the *A. cornea*, thereby determining the specific types and concentrations of melanin in each color variant. These findings validate the argument in this study regarding the correlation between pigment synthesis integrity and phenotypic traits. It is recommended that subsequent research delve deeper into the types and proportions of melanin in white *A. cornea*.

Table 2 presents statistical results for melanin content and edible quality traits of 138 *A. cornea* strains, including mean values, standard deviations, coefficients of variation, extreme values, and medians for each indicator. Additionally, the Kolmogorov–Smirnov (K-S) test (*p*-values all >0.05) confirmed that the data for each trait conformed to a normal distribution.

### 3.2. Correlation Analysis, Principal Component Analysis

**Figure 3:**
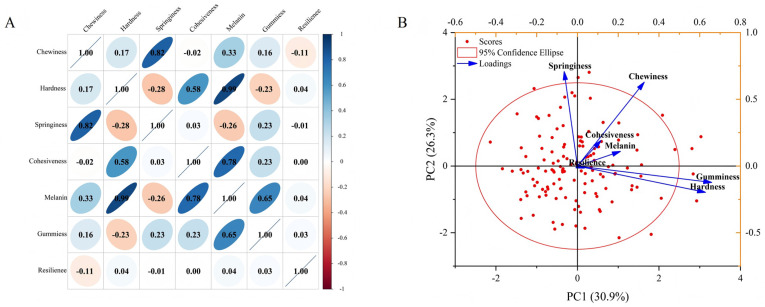
Correlation analysis and principal component analysis of melanin in *A. cornea* and edible quality traits. (**A**) Correlation matrix of melanin content and texture parameters (chewiness, hardness, springiness, cohesiveness, gumminess, resilience). The values shown are Pearson correlation coefficients. Blue indicates positive correlations, red indicates negative correlations, and the color intensity reflects the strength of the correlation. (**B**) The horizontal axis (PC1) represents the first principal component, accounting for 30.9% of the total variance in the original data. The vertical axis (PC2) represents the second principal component, accounting for 26.3% of the total variance in the original data. Red dots: Position of each strain along the PC1 and PC2 principal component dimensions, reflecting strain distribution clusters. Red ellipse: 95% confidence interval, indicating that 95% of strains fall within this range, demonstrating strain concentration trends. Blue arrows: Represent the loadings of the original variables (melanin, hardness, springiness, cohesiveness, gumminess, chewiness, and resilience), in the principal component space. The arrow direction indicates the association between the variable and the principal component, while the arrow length signifies the strength of the association—longer arrows denote stronger correlations.

Pearson correlation analysis results indicate (Figure 3A) that *A. cornea* melanin exhibits a highly significant positive correlation with hardness, a significant positive correlation with cohesiveness and gumminess, and a negative correlation with springiness. In studies of oyster mushrooms, it has also been found that darker strains exhibit greater melanin accumulation in their cell walls and increased cell wall thickness [10], suggesting that melanin content is interrelated with multiple textural attributes in a connected manner. Principal component analysis (PCA) was then conducted to further explore trait variation patterns (Figure 3B). The first two principal components explained 30.9% and 26.3% of the total variance, respectively, accounting for 57.2% cumulatively, and most samples fell within the 95% confidence ellipse. Overall, the PCA biplot summarizes the major variation patterns, with hardness, cohesiveness, gumminess, chewiness, and melanin content grouping in a similar direction, while springiness diverges along the second component. These two components together capture 57.2% of the total variance among the 138 *A. cornea* strains.

### 3.3. SNP Density Analysis

**Figure 4:**
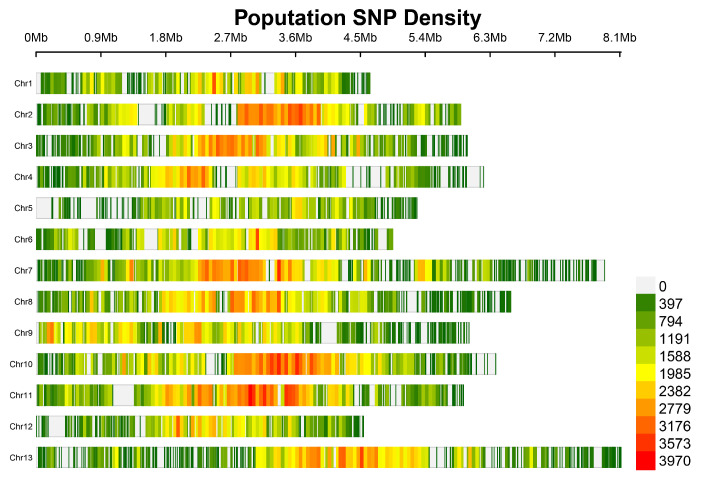
SNP density distribution map. The horizontal axis represents chromosome length in Mb (million base pairs), ranging from 0 Mb to 8.1 Mb. The vertical axis denotes chromosomes 1 through 13 (chr1-chr13), with each row corresponding to the sequence of the respective chromosome. Colors progress from green to yellow to orange to red, indicating increasing density values—that is, the number of SNPs per unit length.

A total of 280 GB of raw sequencing data were generated from 138 *A. cornea* accessions using the Illumina NovaSeq 6000 platform. The average alignment rate of 76.34%, and the mean sequencing depth is 17.42×. GWAS-SNP density calculations were performed for seven traits, including melanin content and edible quality, in 138 *A. cornea* samples. As shown in Figure 4, the SNP density distribution across 13 chromosomes ranged from 0 to 8.1 Mb. The median densities among chromosomes were comparable, with chromosomes Chr2, Chr3, Chr4, Chr7, Chr8, Chr10, Chr11, and Chr13 exhibiting multiple continuous and relatively high SNP density regions, while chromosomes Chr1, Chr5, Chr6, Chr9, and Chr12 exhibited discontinuous SNP density distributions. This reflects variations in genetic diversity across chromosomes. Additionally, we observed fluctuations in SNP density within the 2.2–4.0 Mb range across different segments along individual chromosomes. Orange and red hues indicate higher SNP density, while green hues indicate lower density. These local fluctuations may correlate with functional genomic regions (e.g., coding regions, non-coding regulatory regions), where functionally complex areas often harbor more SNPs. These findings indicate that the genotyping data quality in this study is reliable, providing a solid foundation for subsequent association analyses.

### 3.4. Genome-Wide Association Study and Gene Function Prediction

GWAS was conducted using a mixed linear model (LMM) to analyze the association between melanin content in *A. cornea* and six edible quality traits (hardness, springiness, cohesiveness, gumminess, chewiness, and resilience). Since no associated loci were identified for the resilience trait, it was excluded from subsequent analyses. A total of 15,597,589 SNP loci were detected across 138 *A. cornea*. Screening criteria included LOD scores > 3, regions located within exons (coding regions), and non-synonymous SNVs (mutations causing amino acid substitutions), gene functional annotations (Table S3). The annotation information on melanin and food quality can be found in Supplementary Materials File S2.

**Figure 5:**
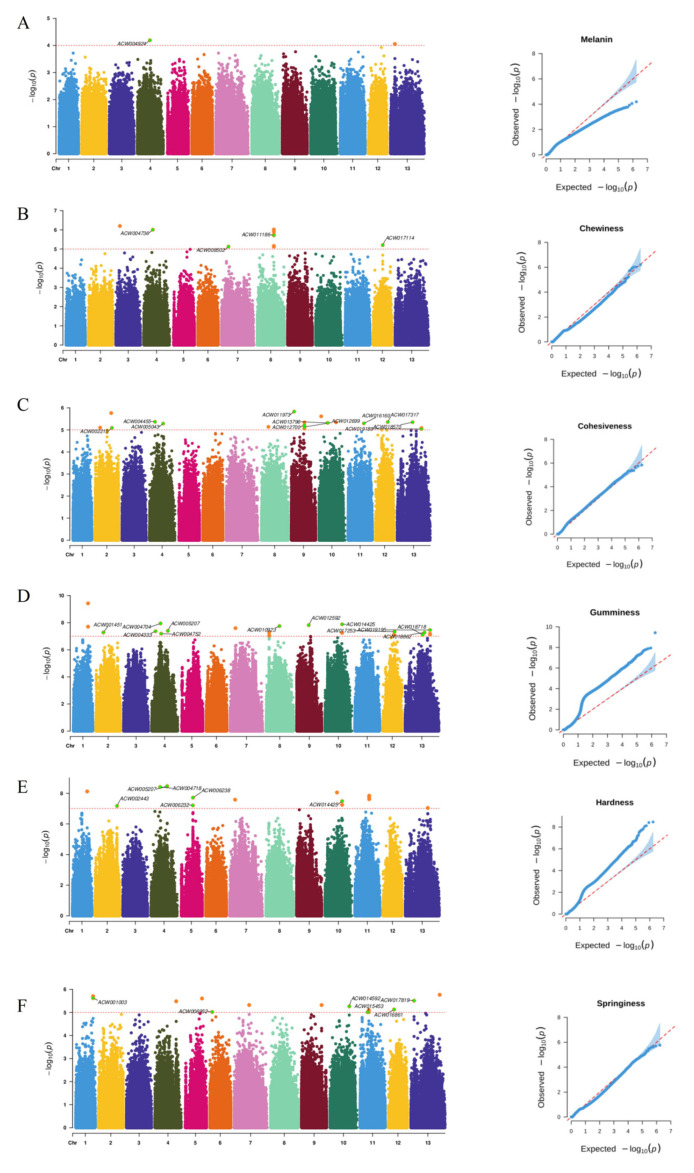
Manhattan plots of melanin content and edible quality in *A. cornea*, along with corresponding QQ plots for each trait. (**A**–**F**): The horizontal axis represents the position of the chromosome, the vertical axis represents the *p*-value after taking −log10, and the red dashed line represents the position of the corresponding threshold after taking −log10. (**A**) Melanin Manhattan plot. (**B**) Chewiness Manhattan plot. (**C**) Cohesiveness Manhattan plot. (**D**) Gumminess Manhattan plot. (**E**) Hardness Manhattan plot. (**F**) Springiness Manhattan plot. Manhattan Plot: The horizontal axis represents chromosome number and position, while the vertical axis displays the −log10 *p*-value (significance). Higher *p*-values indicate stronger significance. To minimize false positives, we set distinct significance thresholds for each trait to identify SNPs with stronger associations. QQ Plot: The horizontal axis represents quantiles from a standard normal distribution, while the vertical axis shows observed quantiles (*p*-values) from the strain data. When blue dots cluster closely around the red diagonal line, the data exhibit a normal distribution.

A total of 35 SNPs were significantly associated with the melanin in *A. cornea* (Figure 5A). *ACW004924*, located on Chr4, is only associated with one SNP locus. BLASTP search against the NCBI non-redundant protein database (parameters and accession versions detailed in the Section 2) revealed that the protein encoded by *ACW004924* shares high sequence similarity with fungal laccases and contains the characteristic copper-binding domains of the polyphenol oxidase (PPO) family. Domain-based annotation further supports its classification within the PPO family and suggests a role in the 1,8-dihydroxynaphthalene (DHN) melanin synthesis pathway. We therefore predict *ACW004924* to encode a laccase, which functions as a redox enzyme catalyzing the oxidation of phenolic compounds into highly reactive quinones—the key initiation step in melanin synthesis in *A. cornea* [2,37,38]. This functional prediction is consistent with findings in Aspergillus fumigatus, where silencing of the laccase gene impairs melanin synthesis and reduces cell wall integrity [39]. In cases where standard genome annotation for *A. cornea* remains incomplete or returns only “hypothetical protein,” the functional assignments made here are based on conserved domain and homology analyses and should be regarded as tentative. *ACW004924* represents a key candidate gene driving melanin synthesis in *A. cornea*.

Regarding chewiness, four SNPs were significantly associated with the trait (Figure 5B): *ACW004736*, *ACW008502*, *ACW011186*, and *ACW017114*. These are located on chromosomes Chr4, Chr7, Chr8, and Chr12, respectively, with each SNP corresponding to one candidate gene. *ACW004736* (Chr4) belongs to the same laccase family as *ACW004924* and is also predicted to function in melanin synthesis. Its standard automated annotation returned “hypothetical protein AURDEDRAFT_85175”; the laccase assignment is based on sequence homology and conserved domain analysis. *ACW008502* (Chr7) is predicted to encode a small molecule transporter that may participate in the transport of cell wall components. The original genome annotation for this locus was “hypothetical protein AURDEDRAFT_112903,” so the proposed function should be regarded as tentative. *ACW011186* (Chr8) is annotated as an alpha/beta-hydrolase and is further presumed to act as a chitinase, likely modifying chitin or wax components in the cell wall of *A. cornea*, thereby influencing toughness and springiness [40]. *ACW017114* (Chr12) encodes a hydrolytic enzyme belonging to the haloalkane dehalogenase superfamily; its initial annotation was “hypothetical protein AURDEDRAFT_149843.” In Saccharomyces cerevisiae, deletion of the orthologous gene YMR318C compromises cell wall integrity and rigidity [41]. It is therefore speculated that *ACW017114* may influence the mechanical properties of *A. cornea* tissue by affecting the cross-linking or metabolism of cell wall polysaccharides.

Regarding cohesiveness, a total of 11 SNPs were significantly associated with the trait (Figure 5C): *ACW002215*, *ACW004455*, *ACW005043*, *ACW011973*, *ACW013796*, *ACW012700*, *ACW012699*, *ACW016160*, *ACW017317*, *ACW018570*, and *ACW019189*. These SNPs are distributed across chromosomes Chr2, Chr4, Chr9, Chr10, Chr11, Chr12, and Chr13. Among them, 2 SNPs are distributed on Chr4, 3 SNPs are distributed on Chr9, and 1 SNP is distributed on each of the remaining chromosomes. Five SNPs are located within or in tight linkage with candidate genes: *ACW018570*, *ACW019189*, *ACW016160*, *ACW012699*, and *ACW005043*, each gene being tagged by one SNP. Through integrative analysis of melanin content and edible quality traits in 138 *A. cornea* strains, we found that cohesiveness is closely linked to melanin deposition and is jointly influenced by two major functional categories: genes involved in pigment synthesis and those responsible for cell wall construction. *ACW018570* (annotated as cytochrome P450) and *ACW019189* (annotated as monooxygenase) are predicted to participate in the melanin biosynthetic pathway, potentially catalyzing oxidative reactions that contribute to pigment deposition in *A. cornea*. In the well-characterized L-DOPA melanin pathway, monooxygenase-type enzymes catalyze the hydroxylation of tyrosine to DOPA, which represents the rate-limiting initial step of melanogenesis in many organisms [42]; similarly, cytochrome P450 enzymes have been implicated in oxidative coupling reactions during melanin polymerization in certain fungi [37,43,44]. However, the pigment in *A. cornea* was previously identified as γ-glutaminyl-3,4-dihydroxy-benzoate (GDHB) synthesized via the shikimate pathway [45], and the precise catalytic roles of these two enzymes within the GDHB melanin pathway remain to be experimentally validated. The remaining three genes are implicated in cell wall remodeling. Their standard automated annotation for *A. cornea* returned only uninformative “hypothetical protein” designations (AURDEDRAFT_189138, AURDEDRAFT_185413, and AURDEDRAFT_47977, respectively); the functional assignments proposed below are therefore based on conserved domain and homology analyses and should be considered tentative. *ACW016160* encodes a putative expansin-like protein, *ACW012699* a putative glycoside hydrolase, and *ACW005043* a putative cystine-rich cell wall protein. Collectively, these three genes are predicted to enhance cell wall strength and toughness by remodeling the polysaccharide network and forming disulfide cross-links, thereby significantly improving fruiting body cohesiveness [6,46,47].

Regarding gumminess, a total of 12 SNPs were significantly associated with the trait (Figure 5D): *ACW001451*, *ACW004704*, *ACW004333*, *ACW005207*, *ACW004752*, *ACW010923*, *ACW012592*, *ACW014425*, *ACW017253*, *ACW019195*, *ACW018718*, and *ACW018862,* located on chromosomes Chr2, Chr4, Chr8, Chr9, Chr10, Chr12, and Chr13. Chr4 contains four SNPs, and Chr13 contains three SNPs. *ACW001451* is predicted to encode a tyrosinase-related protein that functions as a core executor of melanin synthesis, directly catalyzing melanin production. Melanin deposition has been demonstrated to enhance the mechanical integrity of fungal cell walls significantly. *ACW001451* is predicted to encode a tyrosinase-related protein. Based on the well-established role of tyrosinases in catalyzing key oxidative steps in melanin biosynthesis across diverse organisms [48], we hypothesize that *ACW001451* functions as an important enzyme in melanin production in *A. cornea*, although its precise catalytic role in the GDHB pathway remains to be experimentally confirmed. Melanin deposition has been demonstrated to significantly enhance the mechanical integrity of fungal cell walls [49]. This indirectly stabilizes the colloid layer. *ACW010923* is annotated as a bud-site selection protein involved in cell polarity establishment and is predicted to determine the three-dimensional architecture of the colloid layer by regulating hyphal branching [50]. *ACW012592*, a phosphoglycerate transporter, and *ACW005207*, an ATP/GTP-binding protein, respectively supply raw materials and energy for the synthesis of gelatinous substances. Additionally, other genes may participate in the transport of cell wall components and in polysaccharide modification, collectively contributing to the tough, viscoelastic texture of *A. cornea*.

Regarding hardness, six SNPs were significantly associated with the trait (Figure 5E): *ACW002443*, *ACW005207*, *ACW004718*, *ACW006238*, *ACW006232*, and *ACW014425*. These SNPs are located on chromosomes Chr2, Chr4, Chr5, and Chr10. Among them, two SNPs are distributed on Chr4, two SNPs are distributed on Chr5, and one SNP is distributed on each of the remaining chromosomes. Of these, four SNPs are situated within or tightly linked to candidate genes, each tagging a single gene: *ACW002443*, *ACW005207*, *ACW006232*, and *ACW006238*. *ACW002443* is predicted to encode a tyrosinase, and *ACW006232* a laccase (polyphenol oxidase); both are key enzymes in melanin biosynthesis, directly regulating melanin yield and aggregation. *ACW006238* is predicted to encode a chitin synthase, which constitutes the structural scaffold of the cell wall and directly determines the fundamental hardness of the fruiting body. Overexpression of related chitin synthase genes has been shown to significantly increase chitin content in fungal cell walls, synergistically enhancing mechanical strength alongside melanin deposition [6,51]. *ACW005207* is annotated as a P-loop-containing nucleoside triphosphate hydrolase (GTPase) and is co-expressed with cell wall synthesis-related genes, ultimately influencing the texture and hardness of fruiting bodies [52]. Melanin deposition on robust cell walls further reinforces the mechanical strength and hardness of *A. cornea*, indicating that hardness is a complex trait jointly regulated by structural genes and pigment synthesis genes [53]. The synergistic action of the three genes, *ACW002443* (tyrosinase-related protein), *ACW006232* (laccase), and *ACW006238* (chitin synthase), collectively enhances the hardness of *A. cornea* fruiting bodies. It should be noted that for *ACW002443*, *ACW006232*, and *ACW006238*, the original automated genome annotation for *A. cornea* returned only uninformative “hypothetical protein” designations (AURDEDRAFT_121788, AURDEDRAFT_68301, and AURDEDRAFT_126820, respectively). The functional assignments proposed here are therefore based on conserved domain searches and BLASTP homology and should be regarded as tentative.

Regarding springiness, six SNPs were significantly associated with the trait (Figure 5F): *ACW001003*, *ACW006952*, *ACW014592*, *ACW017819*, *ACW015453*, and *ACW016861*. These are distributed across chromosomes Chr1, Chr6, Chr10, Chr11, Chr12, and Chr13, respectively. Of these, four SNPs, *ACW001003*, *ACW006952*, *ACW014592*, and *ACW015453,* are located within candidate genes, each tagging a single gene. The remaining two SNPs (*ACW017819* and *ACW016861*) map to intergenic regions. *ACW001003* is predicted to encode a tyrosinase/laccase and is identified as a direct regulator of melanin biosynthesis; its product enhances the mechanical strength of cell walls. *ACW014592* is annotated as a P-loop-containing nucleoside triphosphate hydrolase (likely a small GTPase) and is predicted to act as a core regulator of cytoskeletal dynamics, directly determining hyphal flexibility and physical springiness. Rho family GTPases have been shown to function as master switches for actin cytoskeletal reorganization, controlling apical growth, branching, and responses to mechanical stress in hyphae [54]. Arkowitz et al. [55] further reported that small GTPases participate in cell separation and cytokinesis and are crucial for cell wall synthesis in filamentous fungi. It is therefore speculated that *ACW014592* may influence edible quality by regulating hyphal flexibility, a finding highly consistent with the conclusions of this study. *ACW006952* is predicted to encode a phospholipid-translocating P-type ATPase that maintains membrane structure and organelle function [38], indirectly supporting the above two processes. *ACW015453* is predicted to encode a hydrolase that may participate in cell wall remodeling, influencing the mechanical strength and springiness of the fruiting bodies of *A. cornea* [56] and indirectly supporting the aforementioned two processes. The *ACW015453* hydrolase may participate in cell wall remodeling to influence the mechanical strength and springiness of the fruiting bodies of *Auricularia heimuer* [6]. These findings have preliminarily established a gene regulatory network, revealing that melanin deposition and cytoskeletal remodeling may be key intrinsic mechanisms jointly influencing the springiness of *A. cornea*. This discovery provides critical targets for subsequent functional validation. *ACW001003* (AURDEDRAT_163416) and *ACW015453* (AURDEDRAT_156016) are hypothesized proteins, and the proposed functions are preliminary predictions.

The homology alignment information of the assumed proteins and key candidate genes involved in the above content is shown in Table S4. Through genome-wide association analysis of melanin content and edible quality traits in *A. cornea*, we identified strong associations between melanin synthesis and multiple texture characteristics (hardness, springiness, chewiness, cohesiveness, and gumminess). The core mechanism involves the melanin synthesis key enzymes lacquerase *ACW004924* and tyrosinase *ACW001451* and *ACW002443*. These key melanin synthesis enzymes not only directly catalyze pigment formation but also synergistically regulate cell wall structure and composition, collectively influencing the physical properties of fruiting bodies. Melanin deposition and cell wall structural genes (chitin synthase *ACW006238*, expansin-like protein *ACW016160*, glycoside hydrolase *ACW012699*) significantly enhance the fruit body’s hardness, cohesiveness, and chewiness. Concurrently, cytoskeletal regulatory proteins (small GTPase *ACW014592*) and cell wall modification enzymes (keratinase *ACW011186*) further modulate springiness and gelatinous texture by influencing hyphal flexibility and cell wall cross-linking. Melanin in *A. cornea* contributes to the construction and reinforcement of the cell wall scaffold by interacting with cell wall components such as chitin and cell wall polysaccharides; this process increases the hardness and reduces the springiness of the fruiting body. The enhanced cohesiveness and adhesion are associated with melanin’s role in promoting intercellular polysaccharide cross-linking or binding to structural proteins, make the fruiting body tissue more compact [37]. Preliminary research by our team identified the PPO family (containing tyrosinase-like functional domains) as key enzymes in pigment synthesis. Velvet family genes AcVeA and AcVelB form a dimer to regulate pigment deposition. Together, these three components establish a molecular chain: upstream regulatory factors (AcVeA-AcVelB) → core catalytic enzymes (laccase/PPO) → pigment synthesis [45]. The tyrosinase-related gene *ACW002443* and laccase genes *ACW004924* and *ACW006232* identified in this study are all downstream effectors at this regulatory level, supplementing the downstream regulatory link in the chain from catalytic enzymes to edible quality. These findings reveal that the edible quality of *A. cornea* is a complex trait co-regulated by the pigment synthesis pathway and cell wall construction network. Multiple genes exhibit pleiotropy or functional complementarity, providing key targets for quality breeding.

The quantile-quantile (QQ) plots for chewiness, gumminess, and hardness GWAS showed that most SNPs followed the expected null distribution, indicating minimal population stratification and reliable association results. However, several SNPs deviated markedly from the diagonal line and exceeded the genome-wide significance threshold (FDR < 0.05). These SNPs were mapped to uncharacterized loci and were not prioritized for further functional analysis in this study. Nevertheless, their strong statistical signals suggest they may tag regulatory elements or unannotated genes that merit investigation in future studies. Comparable patterns of additional significant but uncharacterized GWAS signals have been observed in other fungal association studies [57,58].

### 3.5. Joint Analysis of COG Ortholog Clusters and GO Enrichment Analysis

**Figure 6:**
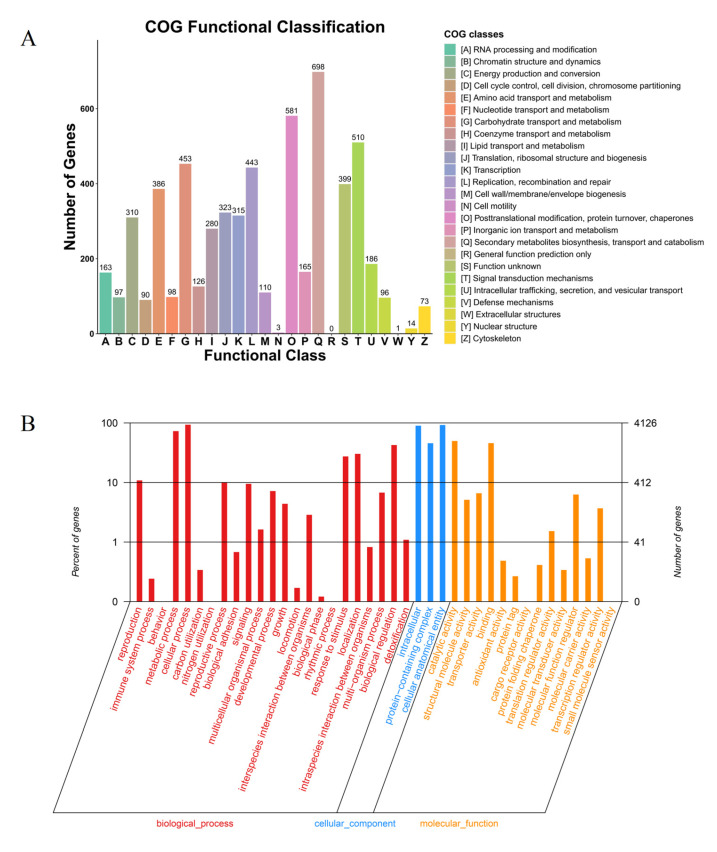
COG ortholog groups and GO enrichment diagram. (**A**) COG ortholog grouping diagram, with the horizontal axis representing functional categories and the vertical axis representing gene counts. (**B**) GO enrichment plot. The bottom section displays classification modules divided into three major categories: red represents biological process, blue represents cellular component, and orange represents molecular function. Each sub-label denotes a specific functional entry. The left vertical axis shows the proportion of genes within a function relative to the total gene count; the right vertical axis indicates the actual number of genes contained within a function.

COG results classify all annotated genes A–Z (Figure 6A). Category O (post-translational modification, protein turnover, molecular chaperones) comprises 698 genes involved in protein folding and modification. These genes may influence the structural stability of cell wall proteins in *A. cornea* (e.g., gelatinous proteins), thereby affecting qualities such as hardness and gumminess. Next is Class M (cell wall/membrane/envelope biogenesis), with 581 genes, indicating the cell wall forms the material basis for *A. cornea* texture (hardness, springiness). Class T (signal transduction mechanisms) contains 510 genes, suggesting strong capabilities in environmental signal perception and intracellular signaling. Category G (carbohydrate transport and metabolism) contains 453 genes. Carbohydrates (such as polysaccharides) constitute the core edible components of *A. cornea*, and their metabolic processes influence traits like chewiness and resilience.

GO enrichment results (Figure 6B) indicate that melanin in *A. cornea* and edible quality primarily encompass three aspects: biological processes, cellular components, and molecular functions. This indicates gene enrichment in metabolic activities, stimulus responses, cellular structures like protein complexes, and molecular functions such as binding and catalytic activity. Among these, binding/catalysis and catalytic activity within molecular functions, along with metabolic processes and cellular processes in biological processes, show relatively prominent gene associations. From the biological process perspective, enrichment in “Metabolic Processes” and “Response to Stimuli” indicates that melanin synthesis is part of metabolic processes. This aligns with Ma et al.’s reported light-dependent regulatory mechanism involving the AcVeA-AcVelB dimer, suggesting environmental signals may indirectly influence edible quality by regulating melanin metabolism [45]. Furthermore, cellular responses to environmental stimuli indirectly modulate the metabolism and synthesis of melanin and texture-related substances (e.g., cell wall polysaccharides, structural proteins) [37]. Ultimately, this indirectly influences edible qualities such as the hardness, resilience, and adhesive strength of *A. cornea*. On the other hand, it also demonstrates that metabolic regulation can promote polysaccharide synthesis, thereby enhancing tissue adhesive strength and resilience. At the cellular component level, the enrichment of “protein-containing complexes” suggests that melanin may participate in the formation of intercellular structural complexes (such as cell wall-associated complexes) in *A. cornea*, aiding in the maintenance of cellular mechanical strength [59], this relates to food qualities such as springiness and chewiness, demonstrating that the stability of cellular structures directly influences tissue springiness and resistance during mastication. At the molecular functional level, the significant enrichment of “catalytic activity” and “binding” reflects the catalytic role of enzymes essential for melanin synthesis (polyphenol oxidase) [60]. On the other hand, this demonstrates that melanin can bind to structural molecules within *A. cornea* cells (such as polysaccharides and structural proteins). This binding stabilizes the cellular structure, thereby influencing edible quality indicators like hardness and cohesiveness. It also explains that enhancing intercellular cohesiveness can improve firmness. Research by Rzepka, Yao, and colleagues revealed that tyrosinase (Tyr1) forms a protein complex with laccase (Lac3), synergistically catalyzing the synthesis of melanin precursors. The ratio of their expression levels determines melanin polymerization, thereby influencing cell wall mechanical strength (hardness increased by 23–35%). This discovery elucidates the molecular chain mechanism of “enzymatic reaction-pigment polymerization-structural reinforcement” [61,62].

In summary, melanin-related genes in *A. cornea* show significant enrichment in molecular functions such as “catalytic activity” and “binding cellular components”, including “protein complexes”, and biological processes encompassing “metabolic processes” and “response to stimuli”. Catalytic activity drives melanin synthesis, binding promotes interactions between melanin and structural cellular molecules (such as polysaccharides and structural proteins), and protein complexes contribute to cellular structural integrity. While metabolic and stimulus response processes regulate the metabolic assembly of texture-related substances. Collectively, these mechanisms mediate the influence of melanin on the edible qualities of *A. cornea*, including hardness, springiness, and chewiness.

### 3.6. KEGG Enrichment Analysis

KEGG enrichment results (Figure S2) indicate that melanin synthesis in *A. cornea* is primarily regulated by pathways involving carbon metabolism, glycosylation, and lipid metabolism. Edible quality traits such as chewiness, cohesiveness, hardness, and springiness are associated with pathways including amino acid metabolism, calcium signaling, cell cycle, and hormone signaling. Adhesion and resilience rely more heavily on pathways such as splicing and glycosylation. These pathways participate in regulating corresponding traits through the high enrichment of differentially expressed genes, with core pathways exhibiting both high enrichment levels and statistical significance. The mannose O-glycosylation pathway participates in the glycosylation modification of intracellular proteins. Modified proteins contribute to cellular structural assembly, enhancing intercellular connectivity and thereby improving the springiness and resilience of *A. cornea*. Pathways such as carbon metabolism, RNA transport, and amino sugar and nucleotide sugar metabolism form the foundation for synthesizing *A. cornea* cell wall components (e.g., chitin, glucan). Metabolic products like amino sugars and nucleotide sugars serve as raw materials for cell wall polysaccharides [37]. The polysaccharide composition and structure of the cell wall directly determine the edible qualities of *A. cornea*, such as hardness, cohesiveness, and chewiness. In other words, the denser the cell wall and the more stable the polysaccharide bonds, the better the hardness, cohesiveness, and chewiness of the *A. cornea* [6].

**Figure 7:**
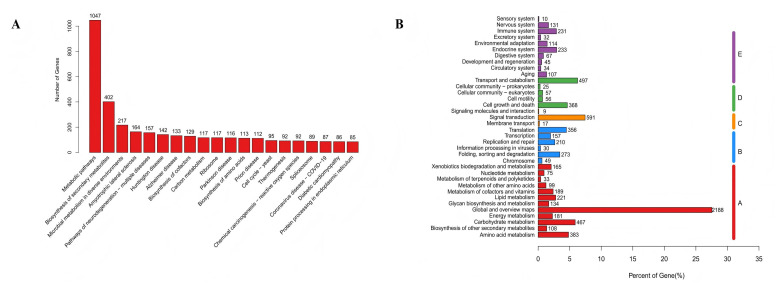
KEGG pathway enrichment plot. (**A**) KEGG pathway gene count bar chart. The vertical axis shows the number of genes contained in the corresponding pathway, while the horizontal axis displays the KEGG pathway name. (**B**) KEGG gene proportion bar chart. The horizontal axis represents the percentage of genes within a KEGG pathway relative to the total number of genes; higher values indicate a greater proportion. The numbers on the bars denote the actual number of genes contained. The vertical axis represents KEGG pathway functions, with A–E corresponding to different KEGG level 1 functional categories.

The gene distribution in KEGG pathways (Figure 7B) reveals that metabolic pathways (Category A) account for the highest proportion. The “Global and overview maps” subcategory, which encompasses broad metabolic overviews, contains the largest number of genes (35%); however, given its generalized nature, this subcategory was excluded from subsequent enrichment-based interpretation to avoid inflated significance. Among the more specific metabolic subpathways, carbohydrate metabolism and amino acid metabolism exhibited the most substantial gene representation. Carbohydrate metabolism and glycan biosynthesis and metabolism provide the biosynthetic machinery for cell wall polysaccharides (e.g., glucan, chitin), whose composition and architecture directly influence the hardness, cohesiveness, and chewiness of *A. cornea* fruiting bodies. Lipid metabolism contributes to intercellular lipid synthesis, affecting cell connectivity and correlating with springiness and resilience. Biosynthesis of other secondary metabolites represents the direct pathway for melanin production in *A. cornea*, and the substantial gene count in this category indicates enrichment of melanin biosynthetic enzymes.

The top 20 KEGG pathways with the highest gene counts were identified (Figure 7A). After excluding “Global and overview maps,” the metabolism pathway (excluding the broad overview subcategory) and biosynthesis of secondary metabolites showed the most pronounced enrichment, followed by pathways with progressively fewer associated genes. This distribution suggests that core metabolic pathways supply precursor molecules (e.g., amino acids and nucleotides) for melanin synthesis, while also supporting the production of cell wall polysaccharides and structural proteins—molecules that directly determine quality traits such as hardness and springiness. The enrichment of biosynthesis of secondary metabolites is consistent with its central role in melanin enzymatic synthesis. Additionally, this study found that melanin enhances mechanical strength by interacting with cell wall components (chitin and polysaccharides). KEGG enrichment in carbon metabolism and polysaccharide biosynthesis pathways further supports a cooperative relationship between pigment synthesis and cell wall assembly. This conclusion aligns with the genomic colinearity analysis by Ma et al. [2], which identified chromosomal structural variations in cell wall synthesis genes within color mutant strains. The association between the GDHB synthesis pathway and cell wall polysaccharide metabolism (carbon metabolism and polysaccharide synthesis) reinforces the model that the coordinated contribution of pigment synthesis and cell wall assembly is a central mechanism underlying the *A. cornea* phenotype (color + texture).

### 3.7. Validation of Key Genes for Melanin and Edible Quality in A. cornea Based on GWAS Screening

Four *A. cornea* strains exhibiting significant variations in melanin content and edible quality were selected: white ACW008, pink ACP126, purple ACP004, and dark reddish-brown ACP022. Six key genes controlling melanin and edible quality were identified: *ACW004924* (laccase), *ACW016160* (expansion-like protein), *ACW006238* (chitin synthase), *ACW014592* (small GTPase), *ACW011186* (keratinase), and *ACW001451* (tyrosinase-related protein). Real-time quantitative PCR (qPCR) validation was performed using the β-tubulin gene (β-TUB) as an internal reference. β-Tubulin was chosen because it is a well-established housekeeping gene, previously validated as stably expressed across different strains and developmental stages in *A. cornea* [24] and widely used as a reliable reference in edible fungi. Primer sequences (Table S5).

**Figure 8:**
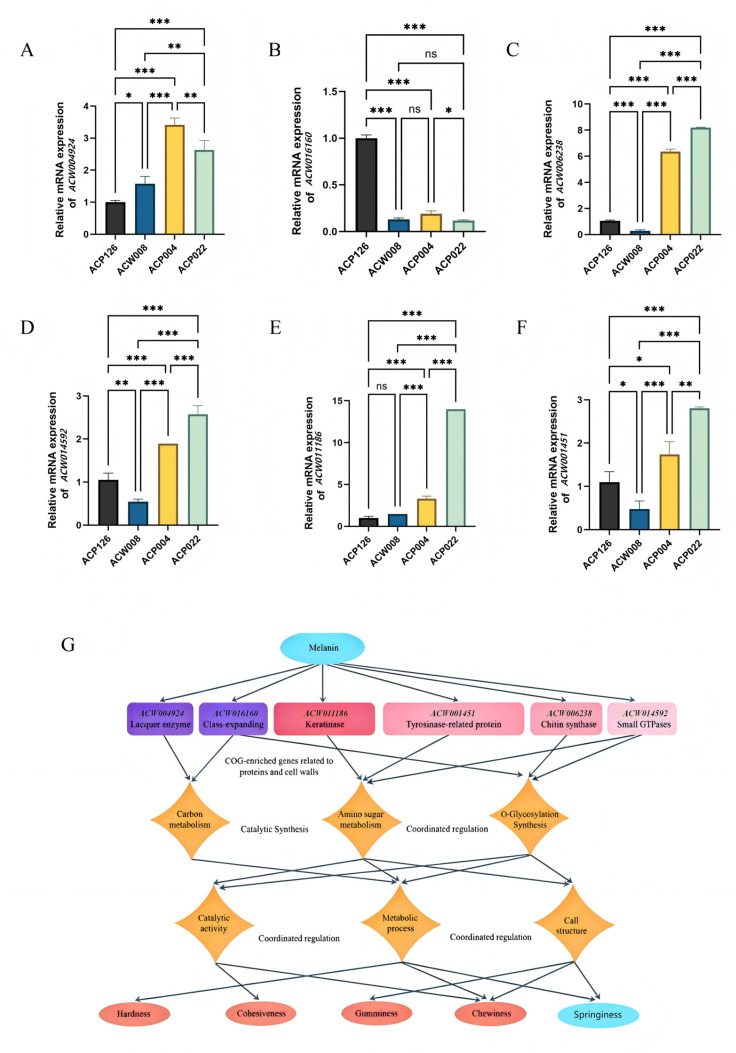
qPCR validation of key genes for melanin and edible quality in *A. cornea*. * *p* < 0.05, ** *p* < 0.01, *** *p* < 0.001, ns (not significant). The horizontal axis represents expression levels, and the vertical axis shows strain names. (**A**) Gene *ACW004924* (laccase). (**B**) Gene *ACW016160* (expansion-like protein). (**C**) Gene *ACW006238* (chitin synthase). (**D**) Gene *ACW014592* (small GTPase). (**E**) Gene *ACW011186* (keratinase). (**F**) Gene *ACW001451* (tyrosinase-related protein). (**G**) Regulatory network diagram of the melanin–gene–pathway–edible quality in *A. cornea*.

Results showed that *ACW004924* (laccase) (Figure 8A) and *ACW001451* (tyrosinase-related protein) (Figure 8F) were significantly more highly expressed in the darker strains (purple and brown) than in the lighter ones (white and pink), suggesting their possible involvement in melanin synthesis. *ACW006238* (chitin synthase) (Figure 8C) showed near-zero expression in the white strain but extremely high expression in purple and brown strains, which may increase chitin content and cell wall density, contributing to a tougher texture. *ACW011186* (keratinase) (Figure 8E) was highly expressed in the brown strain but low in white and pink strains (ns), hinting at a role in cell wall remodeling and melanin deposition. *ACW014592* (small GTPase) (Figure 8D) expression increased with darker pigmentation, potentially mediating melanin and cell wall regulation as a signaling hub. *ACW016160* (expansin-like protein) (Figure 8B) was highest in the pink strain and lower in purple and brown strains, consistent with a looser cell wall and tender texture in pink strains.

Based on these expression patterns, we constructed a hypothetical regulatory network (Figure 8G) linking melanin, genes, pathways, and edible quality. As expression data are correlative, functional confirmation (e.g., gene knockout) is required in future studies.

## 4. Conclusions

This study provides a systematic view of the molecular links between melanin deposition and edible quality in *A. cornea*, delineating a melanin–gene–pathway–quality regulatory network. Melanin was identified as a key determinant of edible quality. Its synthesis is predicted to be initiated by the laccase encoded by *ACW004924*, which catalyzes the oxidation and polymerization of phenolic compounds, thereby promoting melanin deposition. This process is closely coordinated with cell wall assembly: tyrosinase, laccase, and chitin synthase jointly contribute to cell wall scaffold construction and reinforcement, enhancing fruit body hardness, cohesiveness, and gumminess, whereas springiness is influenced by tyrosinase, laccase, and small GTPases that modulate hyphal flexibility. Functional enrichment analysis further indicated that carbon metabolism, amino sugar metabolism, and nucleotide sugar metabolism supply precursors for cell wall polysaccharide biosynthesis. Numerous cell wall-related genes and functional modules—including catalytic activity and metabolic processes—collectively support both melanin synthesis and cell wall assembly. Key functional genes (*ACW004924* laccase, *ACW001451* tyrosinase, *ACW004752* chitin synthase, *ACW011186* chitinase, and *ACW014592* small GTPase) fill critical gaps in the downstream molecular mechanisms connecting pigment synthesis to edible quality, forming a coherent pathway from genetic basis to phenotype. These findings validate and extend our prior work and provide precise molecular targets for the directed improvement of *A. cornea* edible quality through breeding.

## Figures and Tables

**Table 1:** Main instruments and equipment.

Name	Brand	Item Number
Ultra micro spectrophotometer	Baoyude	Micro Drop
Micro high-speed freezing centrifuge	Silo Czech Republic	CF1524R
High-speed tissue grinder	Shanghai Jingxin	Tiss-24
Horizontal electrophoresis tank	Shanghai Tianneng	HE-120
Electrophoresis apparatus	Shanghai Tianneng	EPS-300
UV analyzer	Shanghai Tianheng	ZF-20D
Real-time PCR	ABI	ViiA 7
PCR instrument	Hangzhou lattice	GM-02

**Table 2:** Statistical data on melanin content and edible quality phenotypes of *A. cornea*.

Characteristics	Average	Standard Deviation	Variance	Total	Coefficient of Variation	Minimum Value	Median	Maximum Value	Kurtosis	k-s *p*-Value
Melanin	13.29	3.42	11.72	1754.15	26%	7.51	13.54	19.31	11.80	0.24
Hardness	1274.18	333.00	110,885.85	168,191.28	26%	673.61	1193.26	2342.33	1668.71453	0.33
Springiness	6.08	2.96	8.74	802.27	49%	0.94	5.90515	14.42	13.47638	0.12
Cohesiveness	0.59	0.06	0.01	77.17	11%	0.34	0.59	0.81	0.47	0.26
Gumminess	741.29	199.34	39,736.86	97,850.29	27%	297.13	708.78	1421.02	1123.89	0.12
Chewiness	4406.13	2120.71	4,497,412.46	581,608.61	48%	584.72	4302.42	10,266.31	9681.59	0.22
Resilience	0.62	0.10	0.01	82.40	16%	0.39	0.62	0.97	0.59	0.33

## Data Availability

The original data presented in the study are openly available in NCBI under the accession number PRJNA1406526. The original contributions presented in this study are included in the article/Supplementary Materials. Further inquiries can be directed to the corresponding author.

## Supplementary Materials

The following supporting information can be downloaded at: https://www.mdpi.com/article/10.3390/jof12060381/s1, Figure S1: Pathway functional model of pigment synthesis; Figure S2: KEGG Bubble Enrichment Diagram of Melanin and Edible Quality; Table S1: Strain Information; Table S2: Gene Function Annotation Table; Table S3: Primer Sequences; Table S4: Homology comparison of melanin and food quality related candidate genes in *Auricularia cornea*; Table S5: Primer Sequences; File S1: RNA extraction and cDNA preparation; File S2: Gene annotation data of melanin and food quality.
