# Supplementary material for: Investigating the Genetic and Molecular Basis of Melanin and Edible Quality in Auricularia cornea
Source: J Fungi (Basel). 2026 May 23;12(6):381. doi: 10.3390/jof12060381 (PMC13301874; doi:10.3390/jof12060381)
Supplement: Supplementary file 1 [file jof-12-00381-s001.zip › File S1.pdf]

Detailed steps of RNA extraction

a. take 100 mg of fresh mycelium and grind it into powder with liquid nitrogen.

b. add 1 ml Trizol and place at room temperature for 5 minutes.

c. add 200 μ l chloroform, shake violently for 15 seconds, and place at room temperature for 3 minutes.

d. Centrifuge at 12000 × g for 15 minutes at 4 ° C.

e. transfer the upper aqueous phase to a new centrifuge tube, add 500 μ l isopropanol, mix well, and place at room temperature for 10 minutes.

f. Centrifuge at 12000 × g for 10 minutes at 4 ° C and discard the supernatant.

g. add 1 ml of 75% ethanol to wash the sediment, and centrifuge at 7500 × g at 4 ° C for 5 minutes.

h. discard ethanol, dry at room temperature for 5 minutes, and add 30 μ l RNase free water to dissolve RNA.

Detailed steps of cDNA synthesis

a. take 1 μ g of total RNA, add 1 μ l of oligo (DT) 18 primer, and add RNase free water to 12 μ L.

b. Heat at 65 ° C for 5 minutes and quickly ice bath for 2 minutes.

c. add 4 μ L 5 × reverse transcription buffer, 1 μ l dNTP mixture (10 mm each), 1 μ l RNase inhibitor, 1 μ l reverse transcriptase, and RNase free water to the total volume of 20 μ L.

d. Incubate at 42 ° C for 60 minutes, and heat at 70 ° C for 5 minutes to terminate the reaction.

e. the obtained cDNA was stored at -20 ° C for backup.
