# Supplementary material for: Investigating the Genetic and Molecular Basis of Melanin and Edible Quality in Auricularia cornea
Source: J Fungi (Basel). 2026 May 23;12(6):381. doi: 10.3390/jof12060381 (PMC13301874; doi:10.3390/jof12060381)
Supplement: Supplementary file 1 [file jof-12-00381-s001.zip › Table. S1.pdf]

Strain Information

Number	Strain Number	Type	Source
1	ACW001	Cultivated	Changchun City, Jilin Province
2	ACW003	Cultivated	Changchun City, Jilin Province
3	ACW004	Wild	Yitong City, Jilin Province
4	ACW005	Cultivated	Zhangzhou City, Fujian Province
5	ACW006	Cultivated	Zhangzhou City, Fujian Province
6	ACW008	Wild	Danzhou City, Hainan Province
7	ACW009	Cultivated	Jining City, Shandong Province
8	ACW011	Hybrid	Changchun City, Jilin Province
9	ACW016	Hybrid	Changchun City, Jilin Province
10	ACP001	Cultivated	Hefei City, Anhui Province
11	ACP004	Wild	Nanyang City, Henan Province
12	ACP005	Wild	Hefei City, Anhui Province
13	ACP006	Wild	Hangzhou City, Zhejiang Province
14	ACP007	Wild	Jiaohe City, Jilin Province
15	ACP008	Wild	Jiaohe City, Jilin Province
16	ACP010	Wild	Jiutai City, Jilin Province
17	ACP012	Cultivated	Chengdu, Sichuan Province
18	ACP014	Wild	Zhangzhou City, Fujian Province
19	ACP015	Cultivated	Chengdu, Sichuan Province
20	ACP016	Wild	Yitong Autonomous County, Jilin Province
21	ACP017	Cultivated	Chengdu, Sichuan Province
22	ACP018	Cultivated	Chengdu, Sichuan Province
23	ACP020	Cultivated	Chengdu, Sichuan Province
24	ACP021	Wild	Jiaohe City, Jilin Province
25	ACP022	Cultivated	Jiaohe City, Jilin Province
26	ACP024	Cultivated	Chengdu, Sichuan Province
27	ACP026	Cultivated	Chengdu, Sichuan Province
28	ACP027	Wild	Chengdu, Sichuan Province
29	ACP029	Cultivated	Taian City, Shandong Province
30	ACP030	Cultivated	Zhangzhou City, Fujian Province
31	ACP033	Wild	Zhangzhou City, Fujian Province

Number	Strain Number	Type	Source
32	ACP034	Wild	Zhangzhou City, Fujian Province
33	ACP035	Wild	Hefei City, Anhui Province
34	ACP036	Wild	Taian City, Shandong Province
35	ACP037	Wild	Taian City, Shandong Province
36	ACP038	Wild	Jining City, Shandong Province
37	ACP040	Wild	Zhangzhou City, Fujian Province
38	ACP041	Wild	Zhangzhou City, Fujian Province
39	ACP042	Wild	Hikone City, Japan
40	ACP044	Wild	Hikone City, Japan
41	ACP047	Cultivated	Hikone City, Japan
42	ACP048	Wild	Jiutai City, Jilin Province
43	ACP049	Wild	Bengbu City, Anhui Province
44	ACP050	Wild	Guangzhou City, Guangdong Province
45	ACP051	Wild	Danzhou City, Hainan Province
46	ACP052	Wild	Danzhou City, Hainan Province
47	ACP053	Wild	Danzhou City, Hainan Province
48	ACP054	Wild	Danzhou City, Hainan Province
49	ACP055	Wild	Danzhou City, Hainan Province
50	ACP059	Wild	Okinawa Prefecture, Japan
51	ACP060	Wild	Xuzhou City, Jiangsu Province
52	ACP062	Hybrid	Changchun City, Jilin Province
53	ACP063	Hybrid	Changchun City, Jilin Province
54	ACP064	Hybrid	Changchun City, Jilin Province
55	ACP067	Wild	Haikou City, Hainan Province
56	ACP068	Wild	Hefei City, Anhui Province
57	ACP069	Wild	Lingshui Hainan Autonomous County
58	ACP075	Hybrid	Changchun City, Jilin Province
59	ACP078	Wild	Dudanjiang City, Heilongjiang Province
60	ACP079	Wild	Guiyang City, Guizhou Province
61	ACP080	Wild	Jinan City, Shandong Province
62	ACP081	Wild	Jinan City, Shandong Province

Number	Strain Number	Type	Source
63	ACP082	Wild	Jinan City, Shandong Province
64	ACP083	Wild	Jinan City, Shandong Province
65	ACP084	Wild	Jinan City, Shandong Province
66	ACP085	Wild	Jinan City, Shandong Province
67	ACP086	Wild	Jinan City, Shandong Province
68	ACP087	Wild	Jinan City, Shandong Province
69	ACP088	Wild	Mudanjiang City, Heilongjiang Province
70	ACP089	Wild	Mudanjiang City, Heilongjiang Province
71	ACP090	Wild	Mudanjiang City, Heilongjiang Province
72	ACP091	Wild	Mudanjiang City, Heilongjiang Province
73	ACP092	Wild	Mudanjiang City, Heilongjiang Province
74	ACP093	Wild	Mudanjiang City, Heilongjiang Province
75	ACP094	Wild	Guiyang City, Guizhou Province
76	ACP095	Wild	Guiyang City, Guizhou Province
77	ACP096	Wild	Guangzhou City, Guangdong Province
78	ACP097	Wild	Guiyang City, Guizhou Province
79	ACP098	Wild	Guiyang City, Guizhou Province
80	ACP099	Cultivated	Wuhan City, Hubei Province
81	ACP100	Cultivated	Lingnan City, Guangdong Province
82	ACP101	Cultivated	Wuhan City, Hubei Province
83	ACP103	Wild	Zhengzhou City, Henan Province
84	ACP105	Wild	Zhengzhou City, Henan Province
85	ACP106	Wild	Zhengzhou City, Henan Province
86	ACP107	Wild	Hangzhou City, Zhejiang Province
87	ACP108	Wild	Hangzhou City, Zhejiang Province
88	ACP110	Wild	Hangzhou City, Zhejiang Province
89	ACP111	Wild	Hangzhou City, Zhejiang Province

Number	Strain Number	Type	Source
90	ACP112	Wild	Hangzhou City, Zhejiang Province
91	ACP113	Wild	Hangzhou City, Zhejiang Province
92	ACP114	Wild	Hangzhou City, Zhejiang Province
93	ACP115	Wild	Hangzhou City, Zhejiang Province
94	ACP116	Wild	Hangzhou City, Zhejiang Province
95	ACP117	Wild	Nanjing City, Jiangsu Province
96	ACP118	Wild	Nanjing City, Jiangsu Province
97	ACP119	Wild	Nanjing City, Jiangsu Province
98	ACP120	Wild	Nanjing City, Jiangsu Province
99	ACP121	Wild	Haining City, Zhejiang Province
100	ACP122	Wild	Haining City, Zhejiang Province
101	ACP123	Wild	Haining City, Zhejiang Province
102	ACP124	Cultivated	Suizhou City, Hubei Province
103	ACP125	Cultivated	Gutian County, Fujian Province
104	ACP126	Cultivated	Wangqing County, Jilin Province
105	ACP127	Cultivated	Jining City, Shandong Province
106	ACP129	Wild	Wuhan City, Hubei Province
107	ACP130	Wild	Wuhan City, Hubei Province
108	ACP132	Wild	Wuhan City, Hubei Province
109	ACP133	Wild	Wuhan City, Hubei Province
110	ACP134	Wild	Wuhan City, Hubei Province
111	ACP135	Wild	Wuhan City, Hubei Province
112	ACP136	Wild	Wuhan City, Hubei Province
113	ACP137	Wild	Wuhan City, Hubei Province
114	ACP139	Wild	Hanzhong City, Shaanxi Province
115	ACP140	Wild	Hanzhong City, Shaanxi Province
116	ACP141	Wild	Yibin City, Sichuan Province
117	ACP142	Wild	Yibin City, Sichuan Province
118	ACP143	Wild	Yibin City, Sichuan Province
119	ACP144	Wild	Yibin City, Sichuan Province
120	ACP145	Wild	Yibin City, Sichuan Province
121	ACP146	Wild	Hanzhong City, Shaanxi Province
122	ACP147	Wild	Hanzhong City, Shaanxi Province

Number	Strain Number	Type	Source
123	ACP148	Wild	Hanzhong City, Shaanxi Province
124	ACP150	Wild	Hanzhong City, Shaanxi Province
125	ACP151	Wild	Chengdu, Sichuan Province
126	ACP152	Wild	Lusaka, Zambia, Africa
127	ACP153	Wild	Xianyang City, Shaanxi Province
128	ACP154	Cultivated	Pingdingshan City, Henan Province
129	ACP155	Cultivated	Pingdingshan City, Henan Province
130	ACP156	Wild	Chongqing city
131	ACP157	Wild	Hamilton, New Zealand
132	ACP158	Wild	Hamilton, New Zealand
133	ACP160	Wild	Hamilton, New Zealand
134	ACP162	Wild	Hamilton, New Zealand
135	ACP165	Cultivated	Jinan City, Shandong Province
136	ACP166	Cultivated	Jinan City, Shandong Province
137	ACP167	Cultivated	Shijiazhuang City, Hebei Province
138	ACP170	Cultivated	Shijiazhuang City, Hebei Province
