# Supplementary material for: Investigating the Genetic and Molecular Basis of Melanin and Edible Quality in Auricularia cornea
Source: J Fungi (Basel). 2026 May 23;12(6):381. doi: 10.3390/jof12060381 (PMC13301874; doi:10.3390/jof12060381)
Supplement: Supplementary file 1 [file jof-12-00381-s001.zip › Table. S3.pdf]

Gene Function Annotation Table

| Trait | Gene ID | Chromosome | POS | Pvalue | Name | Function Prediction |
|---|---|---|---|---|---|---|
| Melanin | *ACW004924* | Chr4 | 3004100 | 0.000064332 | hypothetical protein AURDEDRAFT_161151 | Laccase/Polyphenol oxidase |
| | *ACW004736* | Chr4 | 2202926 | 0.0000009768 | hypothetical protein AURDEDRAFT_85175 | Laccase/Polyphenol oxidase |
| | *ACW008502* | Chr7 | 1713161 | 0.00044505 | hypothetical protein AURDEDRAFT_112903 | MFS Transporter |
| Chewiness | *ACW011186* | Chr8 | 4033469 | 0.000049494 | alpha/beta-hydrolase | Keratinase |
| | *ACW017114* | Chr12 | 2357187 | 0.00098077 | hypothetical protein AURDEDRAFT_149843 | Halogenated alkane dehalogenase superfamily hydrolase |
| | *ACW002215* | Chr2 | 4198140 | 0.00098603 | general substrate transporter | MFS Transporter |
| | *ACW004455* | Chr4 | 1350239 | 0.0000043088 | Phosphotyrosyl phosphatase activator | Tyrosine Phosphatase Activator |
| | *ACW005043* | Chr4 | 3360128 | 0.0000051614 | hypothetical protein AURDEDRAFT_47977 | Cystine-rich cell wall proteins |
| | *ACW011973* | Chr9 | 703745 | 0.00052906 | hypothetical protein AURDEDRAFT_115328 | Short-chain dehydrogenase |
| | *ACW013796* | Chr10 | 2152954 | 0.00030649 | NADPH-dependent glutamate synthase | Glutamate synthase |
| | *ACW012700* | Chr9 | 3218692 | 0.00031917 | hypothetical protein AURDEDRAFT_167233 | Acetyl transferase |
| Cohesiveness | *ACW012699* | Chr9 | 3217266 | 0.00076442 | hypothetical protein AURDEDRAFT_185413 | Glycosyl hydrolase family protein |
| | *ACW016160* | Chr11 | 3874729 | 0.0000050542 | hypothetical protein AURDEDRAFT_189138 | Class-expanding protein |
| | *ACW017317* | Chr12 | 3032095 | 0.0000044178 | hypothetical protein AURDEDRAFT_171336 | FAD-conjugated oxidoreductase |
| | *ACW018570* | Chr13 | 3821272 | 0.0000044694 | cytochrome P450 | Cytochrome P450 Monooxygenase |
| | *ACW019189* | Chr13 | 5983326 | 0.00085905 | monooxygenase | FAD-dependent monooxygenase |
| | *ACW001451* | Chr2 | 1823521 | 0.00016013 | hypothetical protein AURDEDRAFT_136539 | Tyrosinase-related protein |
| | *ACW004704* | Chr4 | 2107466 | 0.00066534 | hypothetical protein EXIGLDRAFT_744408 | Glycosyl hydrolase or cell wall modification enzyme |
| Gumminess | *ACW004333* | Chr4 | 920378 | 0.00072616 | hypothetical protein AURDEDRAFT_163867 | ABC Transporter Family |
| | *ACW005207* | Chr4 | 3852231 | 0.00051569 | P-loop containing nucleoside triphosphate hydrolase protein | ATP/GTP-binding protein |
| | *ACW004752* | Chr4 | 2251812 | 0.00096558 | hypothetical protein AcV5_008445 | Chitin synthase |

| Trait | Gene ID | Chromosome | POS | Pvalue | Name | Function Prediction |
|---|---|---|---|---|---|---|
| Hardness | *ACW010923* | Chr8 | 3217178 | 0.00031628 | Bud-site selection protein | Cell Polarity Establishment Protein |
| | *ACW012592* | Chr9 | 2861377 | 0.00000033683 | TPT-domain-containing protein | Glycogen Phosphorylase |
| | *ACW014425* | Chr10 | 4088293 | 0.00092485 | hypothetical protein AURDEDRAFT_87257 | Glycosyltransferase |
| | *ACW017253* | Chr12 | 2825412 | 0.00080911 | unnamed protein product | Unknown |
| | *ACW019195* | Chr13 | 6008144 | 0.00052657 | peptidase S28 | Serine protease |
| | *ACW018718* | Chr13 | 4254163 | 0.00099867 | hypothetical protein AURDEDRAFT_111472 | Short-chain dehydrogenase |
| | *ACW018862* | Chr13 | 4673604 | 0.000011339 | hypothetical protein AURDEDRAFT_111558 | FAD-conjugated oxidoreductase |
| | *ACW002443* | Chr2 | 5275425 | 0.00012664 | hypothetical protein AURDEDRAFT_121788 | Tyrosinase-related protein |
| | *ACW005207* | Chr4 | 3850450 | 0.000011321 | P-loop containing nucleoside triphosphate hydrolase protein | GTPase |
| | *ACW004718* | Chr4 | 2156983 | 0.00092222 | hypothetical protein AURDEDRAFT_150681 | Unknown |
| | *ACW006238* | Chr5 | 3028644 | 0.00096348 | hypothetical protein AURDEDRAFT_126820 | Chitin synthase |
| | *ACW006232* | Chr5 | 3001600 | 0.000048514 | hypothetical protein AURDEDRAFT_68301 | Laccase, polyphenol oxidase |
| | *ACW014425* | Chr10 | 4088757 | 0.00097227 | hypothetical protein AURDEDRAFT_87257 | Unknown |
| | *ACW001003* | Chr1 | 4140481 | 0.0000019772 | hypothetical protein AURDEDRAFT_163416 | Tyrosinase/Laccase or Polyphenol Oxidase Family Proteins |
| Springiness | *ACW006952* | Chr6 | 545019 | 0.000021747 | phospholipid-translocating P-type ATPase | Phospholipid Flipase |
| | *ACW014592* | Chr10 | 4590069 | 0.0000054504 | P-loop containing nucleoside triphosphate hydrolase protein | NTP Hydrolase/Small GTPase |
| | *ACW017819* | Chr13 | 776910 | 0.000015262 | hypothetical protein AURDEDRAFT_163399 | Membrane proteins or enzymes associated with secondary metabolism |
| | *ACW015453* | Chr11 | 1777007 | 0.0000097257 | hypothetical protein AURDEDRAFT_156016 | Hydrolase or carbohydrate-active enzyme |
| | *ACW016861* | Chr12 | 1361871 | 0.0000074771 | hypothetical protein AURDEDRAFT_121997 | Unknown |
