# Supplementary material for: Investigating the Genetic and Molecular Basis of Melanin and Edible Quality in Auricularia cornea
Source: J Fungi (Basel). 2026 May 23;12(6):381. doi: 10.3390/jof12060381 (PMC13301874; doi:10.3390/jof12060381)
Supplement: Supplementary file 1 [file jof-12-00381-s001.zip › Table. S4.pdf]

Homology comparison of melanin and food quality related candidate genes in *Auricularia cornea*

Gene ID	Homologous proteins/families	Matching species	Sequence consistency	E-value	Conservative domain
ACW004924	Laccase-1	*Auricularia heimuer*	85%	0.0	Cu-oxidase_3 (pfam07732)
ACW0011186	Cutinase-like protein	*Exidia glandulosa*	65%	＜1e-100	Abhydrolase_3 (pfam00561)
ACW016160	Expansin-like protein	*Tremella mesenterica*	58%	＜5e-80	DPBB EXPN (cI23764)
ACW001451	Tyrosinase-related protein 1	*Pleurotus ostreatus*	72%	0.0	Tyrosinase (pfam00264)
ACW006238	Chitin synthase 1	*Auricularia polytricha*	90%	0.0	Chitin_synth_1 (pfam01644)
ACW014592	Ras-like GTPase Rab11	*Ganoderma leucocontextum*	82%	0.0	Ras (pfam00071)
ACW004736	Laccase (multi-copperoxidase)	*Trametes versicolor*	58%	2e-128	Cu-oxidase(PF00394),Cu-oxida se_2 (PF07731),Cu-oxidase_3 (PF07732)
ACW008502	MFS transporter	*Aspergillus nidulans*	45%	8e-45	Sugar_tr (PF00083),MFS_1 (PFO7690)
ACW017114	Haloalkane dehalogenase	*Rhodococcus erythropolis*	52%	3e-67	Hydrolase_4 (PF00561),a/B-hydrolase fold(IPR029058)

ACW016160	Expansin-like protein	*Arabidopsis thalianaEXLA1*	39%	5e-38	DPBB domain(IPR007112), RlpA-likedouble-psi beta-barrel(IPR009009)
ACW012699	Glycoside hydrolasefamily 13	*Stemphylium lycopersici*	61%	1e-95	Six-hairpin glycosidase(IPR008928),oligo-1,6-glucosidase(IPR010401)
ACW005043	Cysteine-rich cell wallprotein	*Neurospora crassa*	42%	4e-29	Fungal cell wall proteindomain
ACW002443	Tyrosinase	*Agaricus bisporus*	63%	3e-110	Tyrosinase (PF00264,di-copper center)
ACW006232	Laccase / Polyphenoloxidase	*Trametes versicolor*	60%	1e-135	Cu-oxidase(PF00394),Cu-oxidase_2 (PF07731),Cu-oxidase_3 (PF07732)
ACW001003	Tyrosinase/Laccase(copper oxidase)	*Pleurotus ostreatus*	57%	6e-88	Cu-oxidase (PF00394),AAA_16 (PF13191)
ACW015453	Glycoside hydrolase(putative)	*Trichoderma reesei*	48%	9e-41	Glyco_hydro (variousfamilies, determined by search)
