# Supplementary material for: Investigating the Genetic and Molecular Basis of Melanin and Edible Quality in Auricularia cornea
Source: J Fungi (Basel). 2026 May 23;12(6):381. doi: 10.3390/jof12060381 (PMC13301874; doi:10.3390/jof12060381)
Supplement: Supplementary file 1 [file jof-12-00381-s001.zip › Table. S5.pdf]

Primer Sequences

1-ACW004924-F	ATTGGCAAGCACGAAGAT
1-ACW004924-R	TTCAGCACAACGACCATC

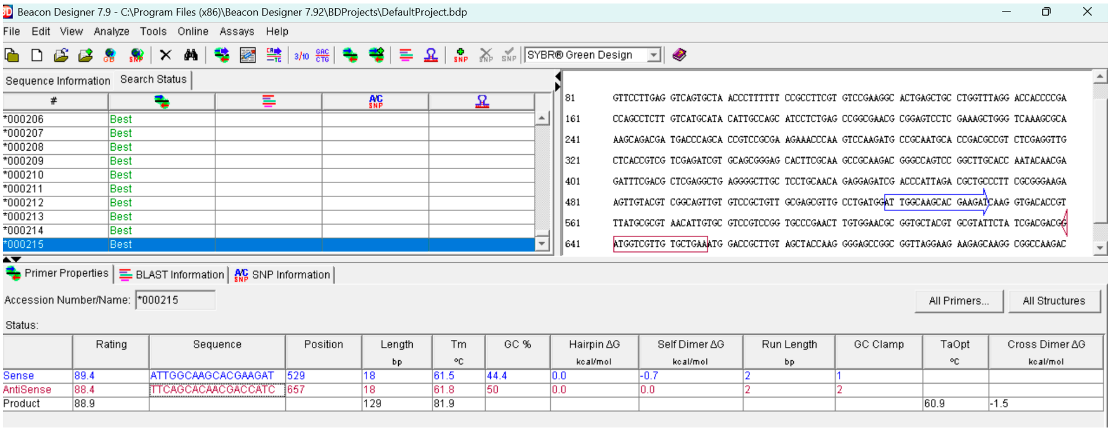

2-ACW011186-F	GCTGTATGCGATAATGTC
2-ACW011186-R	GCTTACCATCTTCAACTG

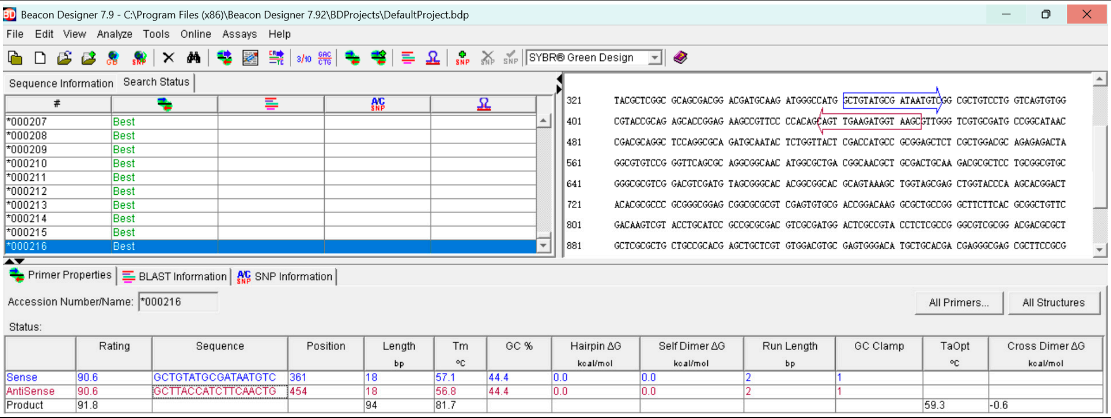

3-ACW016160-F	ATAAGACCGAGGCGTTCC
3-ACW016160-R	GACAGTGGCGACGAAGAT

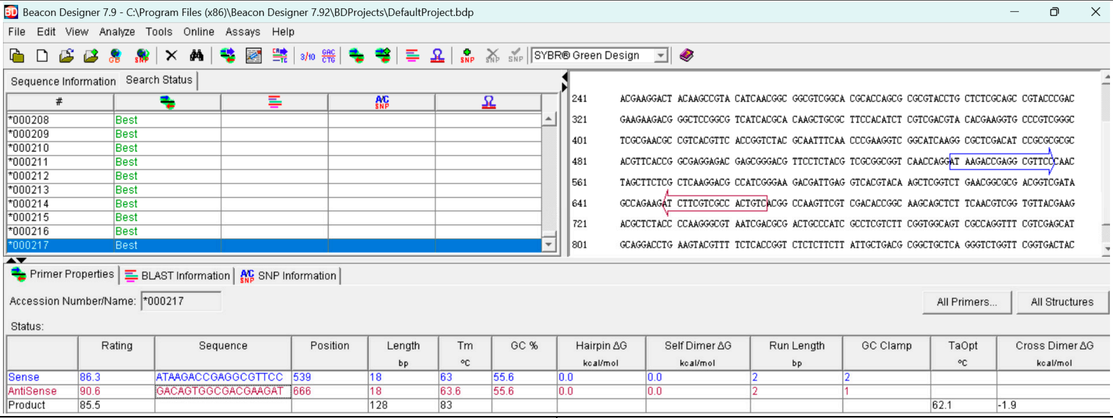

4-ACW001451-F	CCTTCTTGCCGCATCTAC
4-ACW001451-R	GCTTCTCCTCTGGGTTCT

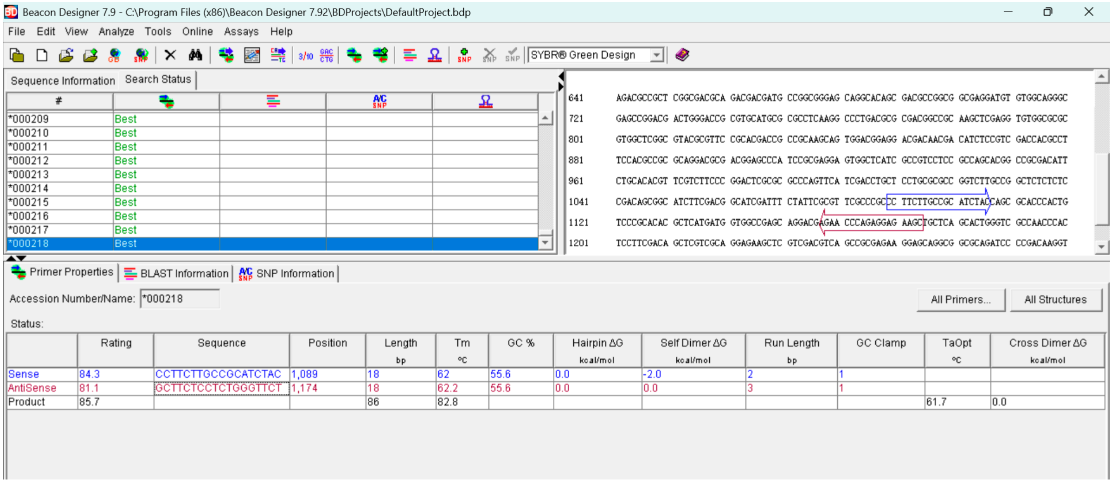

5-ACW006238-F	GCCAACGGTTATTAGGTT
5-ACW006238-R	GGTGATTGTAAGAGGAAGAG

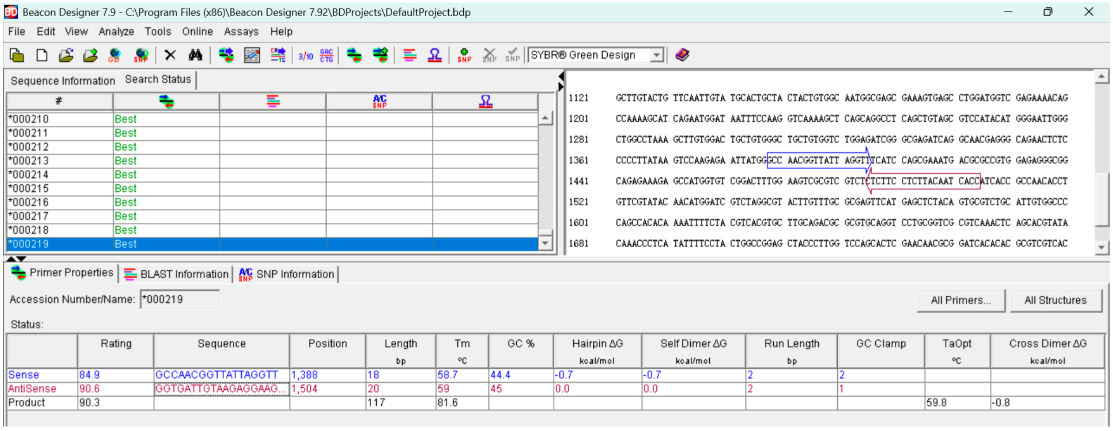

6-ACW014592-F	GTGTTCAGTGTCCAGGAT
6-ACW014592-R	ATGATACGGTAAGCCTCG

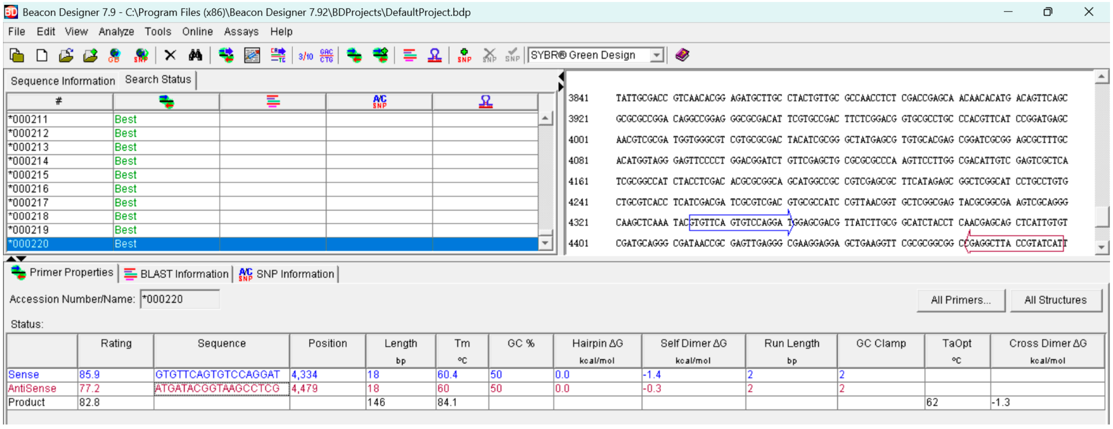

β -TUB -F	TCGCCCAAGGTTTCGGACACT
β -TUB -R	GACGGTTGAGGTACAGACACGA
