# Supplementary figures and images for: Investigating the Genetic and Molecular Basis of Melanin and Edible Quality in Auricularia cornea
Source: J Fungi (Basel). 2026 May 23;12(6):381. doi: 10.3390/jof12060381 (PMC13301874; doi:10.3390/jof12060381)

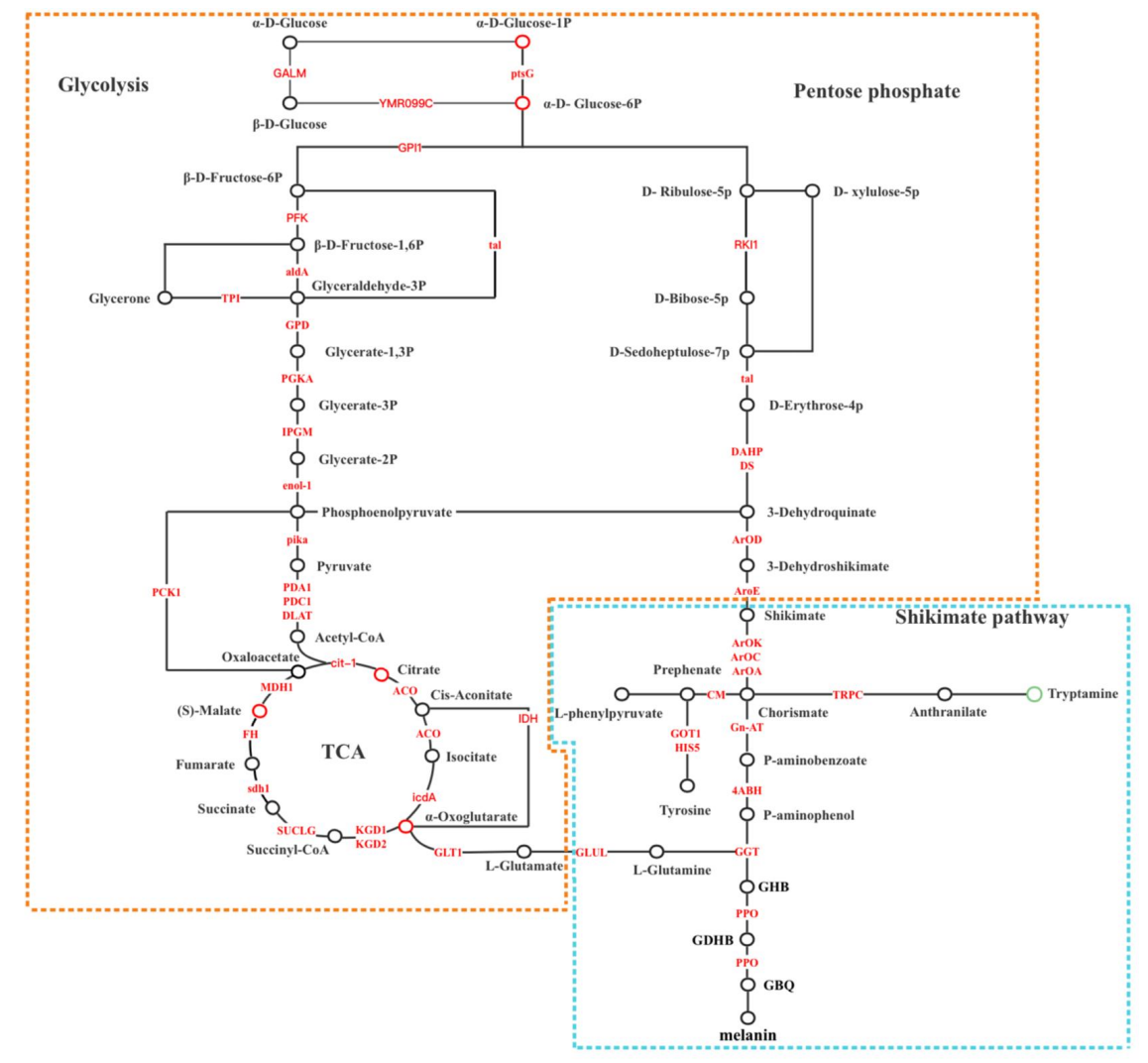

Supplement: Supplementary file 1 [file jof-12-00381-s001.zip › Figure S1.png]

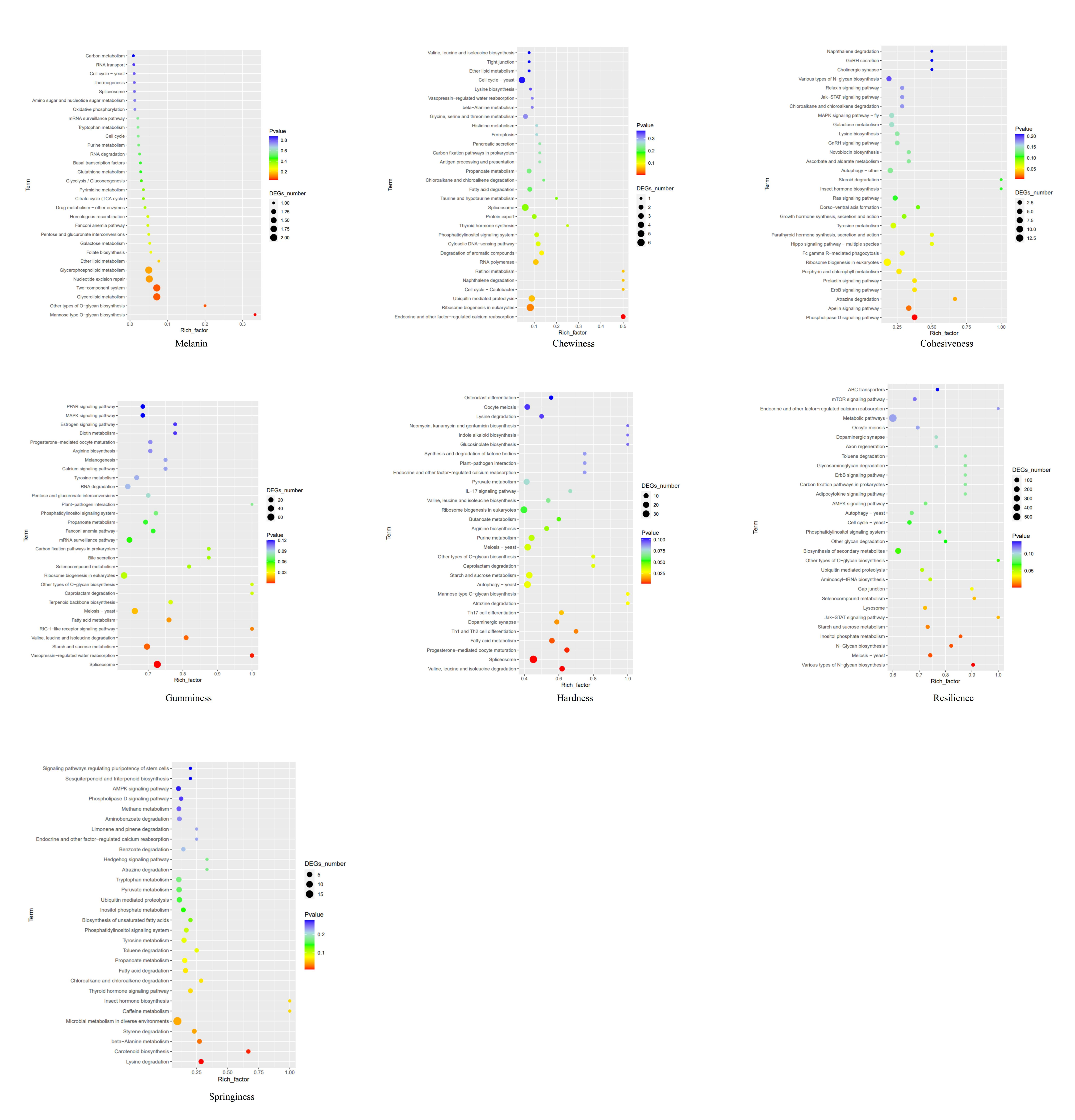

Supplement: Supplementary file 1 [file jof-12-00381-s001.zip › Figure S2.jpg]
